# The potential of natural products to inhibit abnormal aggregation of α-Synuclein in the treatment of Parkinson’s disease

**DOI:** 10.3389/fphar.2024.1468850

**Published:** 2024-10-23

**Authors:** Kaixia Yang, Zhongyue Lv, Wen Zhao, Guogang Lai, Cheng Zheng, Feiteng Qi, Cui Zhao, Kaikai Hu, Xiao Chen, Fan Fu, Jiayi Li, Guomin Xie, Haifeng Wang, Xiping Wu, Wu Zheng

**Affiliations:** ^1^ Department of Neurology, The Affiliated Lihuili Hospital of Ningbo University, Ningbo, Zhejiang, China; ^2^ Neuroscience Medical Center, Ningbo Medical Center Lihuili Hospital, Ningbo University, Ningbo, Zhejiang, China

**Keywords:** Parkinson’s disease, α-Synuclein, natural products, aggregation, misfolding

## Abstract

Parkinson’s disease (PD), as a refractory neurological disorder with complex etiology, currently lacks effective therapeutic agents. Natural products (NPs), derived from plants, animals, or microbes, have shown promising effects in PD models through their antioxidative and anti-inflammatory properties, as well as the enhancement of mitochondrial homeostasis and autophagy. The misfolding and deposition of α-Synuclein (α-Syn), due to abnormal overproduction and impaired clearance, being central to the death of dopamine (DA) neurons. Thus, inhibiting α-Syn misfolding and aggregation has become a critical focus in PD discovery. This review highlights NPs that can reduce α-Syn aggregation by preventing its overproduction and misfolding, emphasizing their potential as novel drugs or adjunctive therapies for PD treatment, thereby providing further insights for clinical translation.

## 1 Introduction

Parkinson’s disease (PD) is a complex progressive neurodegenerative disorder characterized by motor and non-motor symptom (NMS), which may result from genetic and environmental factors. Both genetic and sporadic PD have the loss of dopamine (DA) neurons in substantia nigra (SN) and the presence of Lewy bodies (Bloem et al., 2021; Moore et al., 2005). The primary clinical features include classic dyskinesia symptoms caused by DA loss in the basal ganglia, such as bradykinesia, muscle rigidity, resting tremor, postural instability, and speech disorders. Additionally, NMS arises from the involvement of multiple brain areas and includes swallowing difficulties, drooling, cognitive and emotional disturbances, gastrointestinal dysfunction, and sleep disorders, which appears at all stages of the disease. Although diagnosis primarily focuses on motor symptoms, NMS is also significant. Studies have shown that these symptoms can persist for over 10 years before dyskinesia appears, leading to a decline in patients’ quality of life, with many severely affected by these NMS (Postuma et al., 2012a; Schapira et al., 2017; Balestrino and Martinez-Martin, 2017).

PD is currently the second most common age-dependent neurodegenerative disorder. By 2040, the number of PD patients is expected to reach 17 million, suggesting a “PD pandemic” (Dorsey et al., 2018). Despite decades of research, PD remains incurable (Bloem et al., 2021). Current treatments, including levodopa, DA agonists, catechol-O-methyltransferase (COMT) inhibitors, monoamine oxidase-B (MAO-B) inhibitors, and anticholinergics, only alleviate symptoms without slowing disease progression. Additionally, their effectiveness diminishes over time (Fox et al., 2018). These medications can cause significant clinical side effects, such as motor complications, cognitive and emotional disturbances, psychiatric disorders, and adverse neurological reactions (Bonifácio et al., 2007; Thanvi and Lo, 2004). Therefore, there is an urgent need to develop new therapies that can effectively slow the progression of debilitating motor symptoms.

PD can manifest in two forms: familial/genetic and sporadic. Familial/genetic PD is closely associated with gene mutations, inherited through autosomal dominant or recessive patterns. Mutations in genes such as *SNCA*, *LRRK2*, *VPS35*, *EIF4G1*, and *DNAJC13* are linked to autosomal dominant PD, while *PRKN*, *PINK1*, and *DJ-1* mutations are associated with autosomal recessive forms. Familial/genetic PD typically presents at an earlier age and progresses more rapidly, with Parkin mutations being the most common genetic factor in familial cases. However, the majority of PD cases are sporadic, with no definitive causative factors identified. Lifestyle, adverse environmental exposures, and advanced age are believed to be closely related to the onset of sporadic PD (Costa et al., 2022). For example, a history of psychiatric disorders like anxiety or depression, pesticide exposure, head trauma, rural living, beta-blocker use, and consumption of well water can increase the risk of developing PD. Conversely, factors like smoking, coffee consumption, the use of non-steroidal anti-inflammatory drugs (NSAIDs) and calcium channel blockers, and alcohol consumption are negatively correlated with PD incidence (Noyce et al., 2012). While PD caused by gene mutations is relatively rare, both familial/genetic and sporadic forms present with classic symptoms of bradykinesia, rigidity, and resting tremor. Both forms can also manifest atypical NMS, with neuropsychiatric symptoms potentially more prevalent in familial/genetic PD (Chao et al., 2015).

The hallmark pathological feature of PD is the degeneration of dopaminergic neurons in the SN and striatum. The SN is typically the most severely affected region, with moderate to severe DA neurons loss in this area potentially contributing to the development of bradykinesia. Moreover, the loss of DA neurons extends beyond the SN, affecting multiple brain regions in cases with longer disease duration. These regions include the basal ganglia, hypothalamus, locus coeruleus, medullary tegmentum, hippocampus, temporal lobe, and the pons (Dickson, 2012).

Another crucial pathological feature of PD is the deposition of Lewy bodies, primarily composed of insoluble aggregates formed by misfolded α-Synuclein (α-Syn). The α-Syn aggregation hypothesis has gained significant attention in recent years. Notably, the toxic intermediate oligomers and protofibrils formed during aberrant α-Syn aggregation are particularly detrimental, with soluble protofibrils exhibiting greater toxicity than insoluble mature fibrils, ultimately leading to neurotoxicity (Lashuel et al., 2013; Lücking and Brice, 2000). These toxic intermediates can trigger a cascade of events, including mitochondrial dysfunction, neuroinflammation, neuronal deformation, and ferroptosis, all of which are closely linked to the pathogenesis of PD. Furthermore, they can disrupt synaptic transmission, impair organelle function and cytoskeleton integrity, compromise membrane structure, and disrupt the blood-brain barrier (BBB) (Pacheco et al., 2015; Burré et al., 2018).

The α-Syn aggregates primarily propagate and spread in a “prion-like” manner. Additionally, they can disseminate through tunneling nanotubes (Abounit et al., 2016), exosomes (Grey et al., 2015), and other mechanisms, leading to the widespread deposition of these pathological proteins throughout the brain, particularly in the neocortex, hippocampus, striatum, thalamus, and cerebellum (Burré et al., 2018). Importantly, the abnormal aggregation of α-Syn is not confined to the brain, it has also been observed in the spinal cord and peripheral nervous system, including the sympathetic ganglia, vagus nerve, cardiac nerves, and gastrointestinal system (Beach et al., 2010).

The autophagy-lysosome pathway (ALP) and the ubiquitin-proteasome system (UPS) are the two primary mechanisms for degrading misfolded and aggregated α-Syn proteins. In addition to aggregation driven by structural changes, functional impairments in these degradation pathways contribute to a reduced clearance rate of α-Syn aggregates (Senkevich and Gan-Or, 2020). Interestingly, α-Syn aggregates can also exert inhibitory effects on their own degradation pathways, creating a vicious cycle of aggregation due to their continuous formation, accumulation, and impaired clearance (Yang B. et al., 2023; Park et al., 2023). Therefore, targeting the inhibition of aberrant α-Syn aggregation and fibrillation, along with promoting the breakdown of existing aggregates to mitigate cellular toxicity, represents a promising therapeutic strategy for PD. To date, a limited number of drugs have been developed with this approach, including Anle138b (patent: WO2010000372) (Wagner et al., 2013), NPT200-11 (patent: CN102725284) (Price et al., 2018), and UCB0599 (the R-enantiomer of NPT200-11) (patent: CN110198938) (Smit et al., 2022). These aggregation inhibitors have demonstrated neuroprotective properties in related trials, suggesting that targeting α-Syn aggregation is a viable therapeutic avenue for PD.

Natural products (NPs) are increasingly recognized as important and valuable resources. To date, the development of NPs as emerging therapeutic agents remains a significant area of research in disease treatment, particularly for neurodegenerative disorders like PD. Extensive research has shown the potential of NPs to modulate oxidative stress and mitochondrial damage in PD, with significant discoveries emerging from natural plant-derived products. However, there is a relative paucity of research focusing on the potential of NPs to inhibit α-Syn aggregation, a key pathological hallmark of PD. Therefore, this review aims to bridge this gap by presenting evidence supporting the role of NPs in PD treatment through the inhibition of α-Syn aggregation. The findings discussed herein highlight promising lead compounds for the future development of novel α-Syn-targeted therapeutics and lay a foundation for further exploration of NPs as a therapeutic avenue for PD intervention.

## 2 The structure and aggregation of α-Syn

### 2.1 Structure and aggregation

**FIGURE 1:**
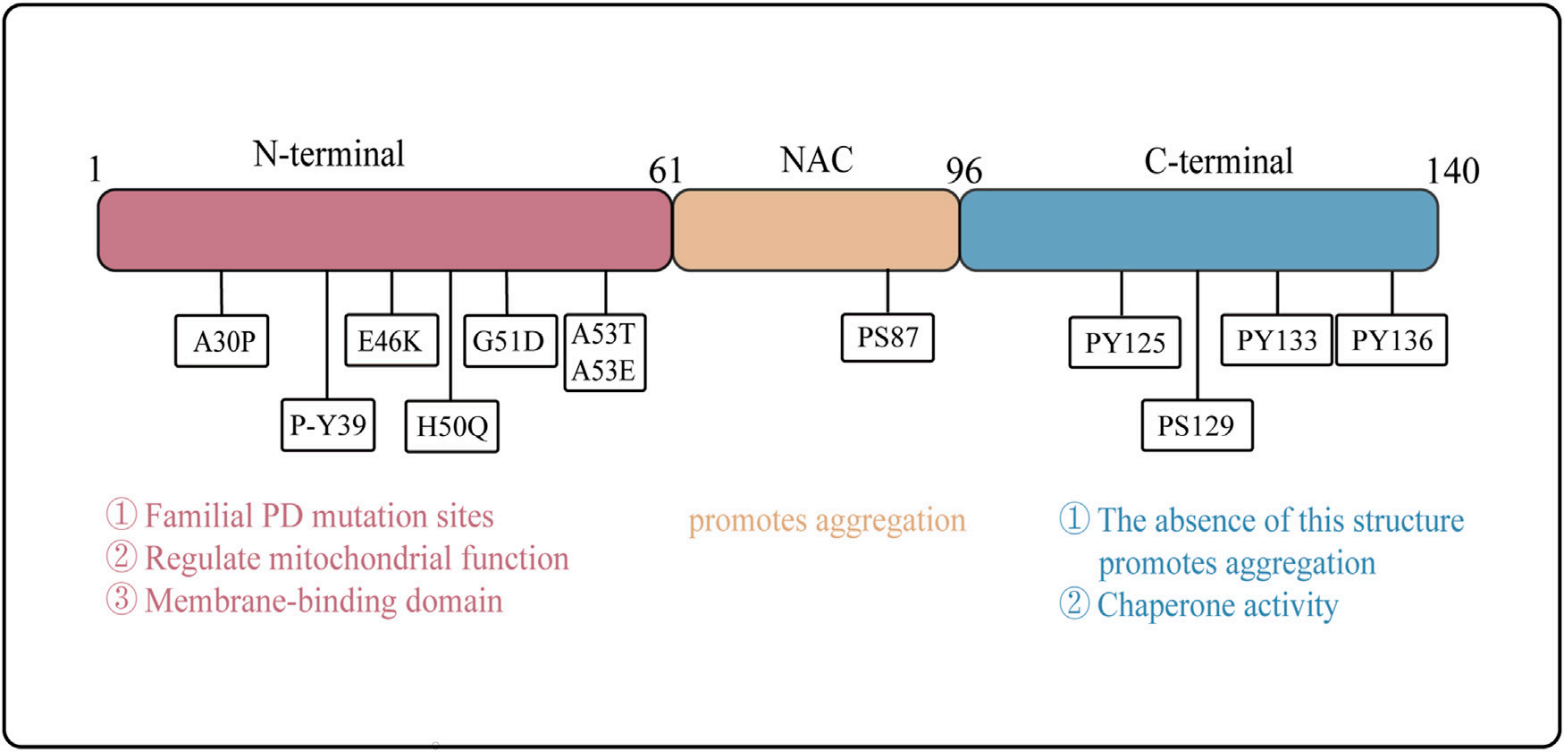
Structure and Function of α-Syn Domains. The structural of α-Syn is illustrated, emphasizing the N-terminal region (residues 1–60), the NAC region (residues 61–95), and the C-terminal region (residues 96–140). Arrows indicate the sites of familial Parkinson’s disease mutations. The distinct functions of the N-terminal, NAC region, and C-terminal domains are delineated, highlighting their respective roles in the protein’s overall activity and pathology.

α-Syn is a small, highly aggregation-prone protein composed of 140 amino acids. It can be divided into three distinct regions: the N-terminal region (residues 1–60), the nonamyloid-β component (NAC) region (residues 61–95), which forms the non-amyloid β component of amyloid plaques, and the C-terminal region (residues 96–140) (Burré et al., 2018). Notably, mutations in the α-Syn gene are linked to the development of familial PD, with A30P, E46K, and A53T being the most extensively studied mutation sites (Polymeropoulos et al., 1997; Krüger et al., 1998; Zarranz et al., 2004) (Figure 1).

(1) N-terminal region: This part of α-Syn is rich in amphipathic amino acid residues and contains seven imperfect repeat sequences along with a conserved sequence KTKEGV. This conserved sequence plays a role in mitochondrial function regulation and has been implicated in mitochondrial dysfunction (Hui, 2020; Burré et al., 2018). Acetylation of the N-terminal can enhance its affinity for lipid membranes (LM) and stabilize its helical structure (Figure 1). Many α-Syn mutation sites reside within the N-terminal region, leading to varying degrees of alteration in membrane binding affinity and aggregation propensity. For instance, the A30P, G51D, and A53E mutations decrease LM affinity, while the E46K mutation increases it. Additionally, the A30P, E46K, and A53T mutations may promote α-Syn aggregation, whereas the G51D mutation hinders it (Dikiy and Eliezer, 2014). Upon binding to LMs, the N-terminal structure of α-Syn undergoes a conformation change from a random coil to a helical structure (Davidson et al., 1998).
(2) NAC region: This region of α-Syn was first identified in the brain senile plaques of Alzheimer’s disease patients. This region exhibits strong hydrophobicity and is crucial for the stabilization and aggregation of α-Syn (Han et al., 1995; Uéda et al., 1993). The NAC region encompasses the minimal sequence necessary for α-Syn aggregation. However, recent studies suggest that specific sequences outside the NAC region also influence α-Syn aggregation, indicating that multiple regions may work in concert to regulate this process (Tripathi, 2020; El-Agnaf and Irvine, 2002) (Figure 1).
(3) C-terminal region: This part of α-Syn is rich in acidic amino acid residues, particularly glutamic acid, enabling it to interact with a diverse array of proteins. Post-translational modifications of α-Syn, including phosphorylation, acetylation, ubiquitination, glycosylation, and nitration, likely play critical roles in regulating its misfolding, abnormal aggregation, and neurotoxicity (Burré et al., 2018). Furthermore, the C-terminal structure is essential for the chaperone activity of α-Syn. Notably, the deletion of the C-terminal region abolishes this chaperone activity, thereby promoting α-Syn aggregation (Kim et al., 2002) (Figure 1).

**FIGURE 2:**
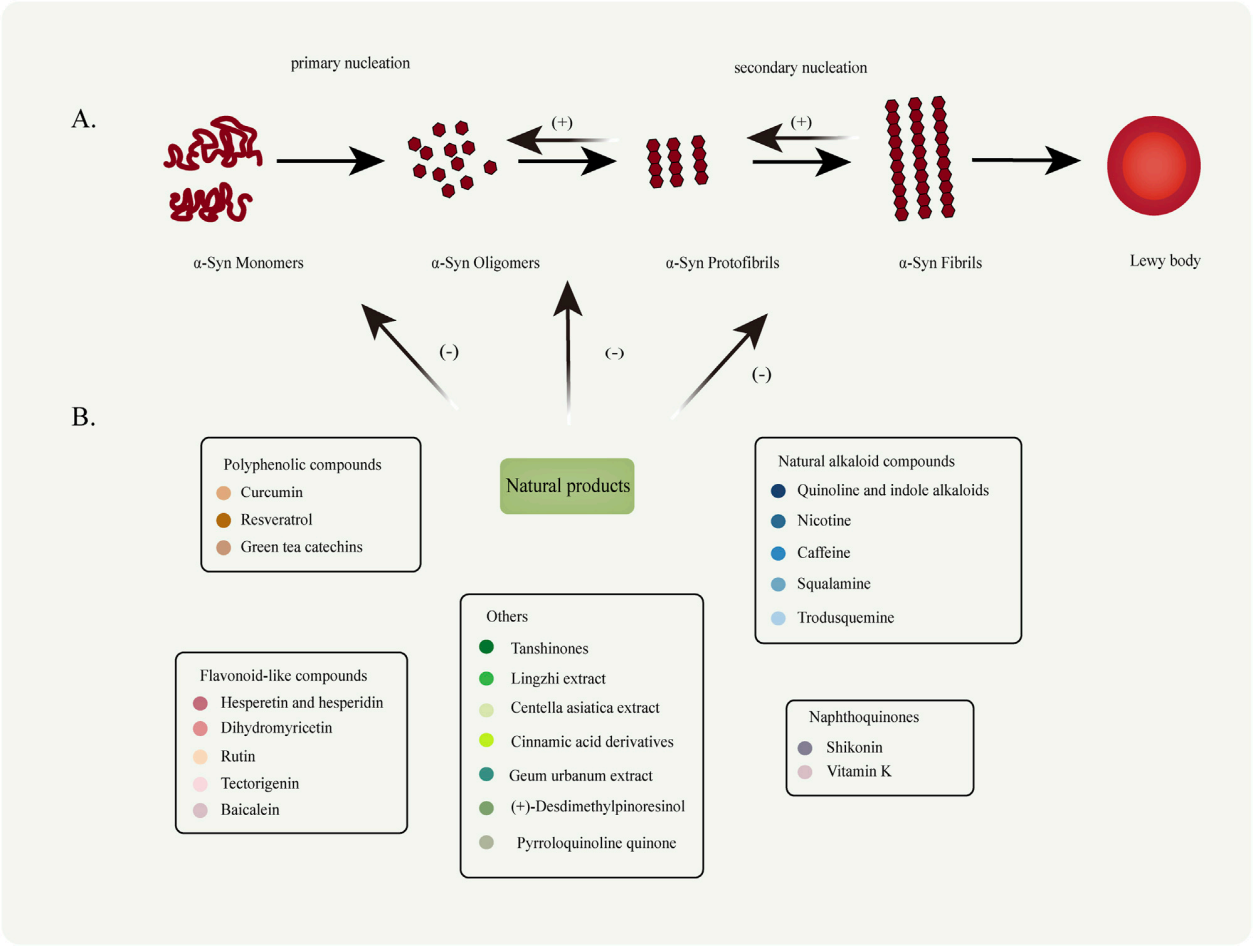
**(A)** Under pathological conditions, naturally disordered α-Syn monomers abnormally aggregate to form oligomers (primary nucleation). These oligomers subsequently extend to generate protofibrils and mature fibrils (secondary nucleation). **(B)** Various types of NPs can inhibit the conversion of α-Syn monomers into oligomers, thereby suppressing oligomer fibrillation and preventing fibril formation. Furthermore, NPs facilitate the degradation of fibrillar products.

α-Syn aggregation follows the general kinetics of protein aggregation, encompassing distinct phases: nucleation (lag phase), elongation, growth, aggregation/fibrillation, and equilibrium/saturation (Xiong et al., 2010; Almeida and Brito, 2020). In solution, α-Syn exists primarily as an unstructured random coil. However, it adopts α-helical structures upon binding to lipids and forms β-sheet-rich structures during aggregation. Under normal physiological conditions, α-Syn exists predominantly in monomeric or tetrameric forms, characterized by stable helical structures that confer resistance to aggregation (Bartels et al., 2011; Burré et al., 2018; Xiong et al., 2010). However, under pathological conditions, α-Syn undergoes misfolding, leading to conformational changes that drive abnormal aggregation. This process results in the formation of various species, including oligomers, protofibrils, and mature aggregates or fibrils, all of which can be cytotoxic to neurons and contribute to neuronal death. Notably, recent studies suggest that intermediate α-Syn oligomers, formed during the aggregation process, may exhibit greater toxicity than mature aggregates and fibrils (Lücking and Brice, 2000) (Figure 2A).

### 2.2 The impact of α-Syn aggregation

**FIGURE 3:**
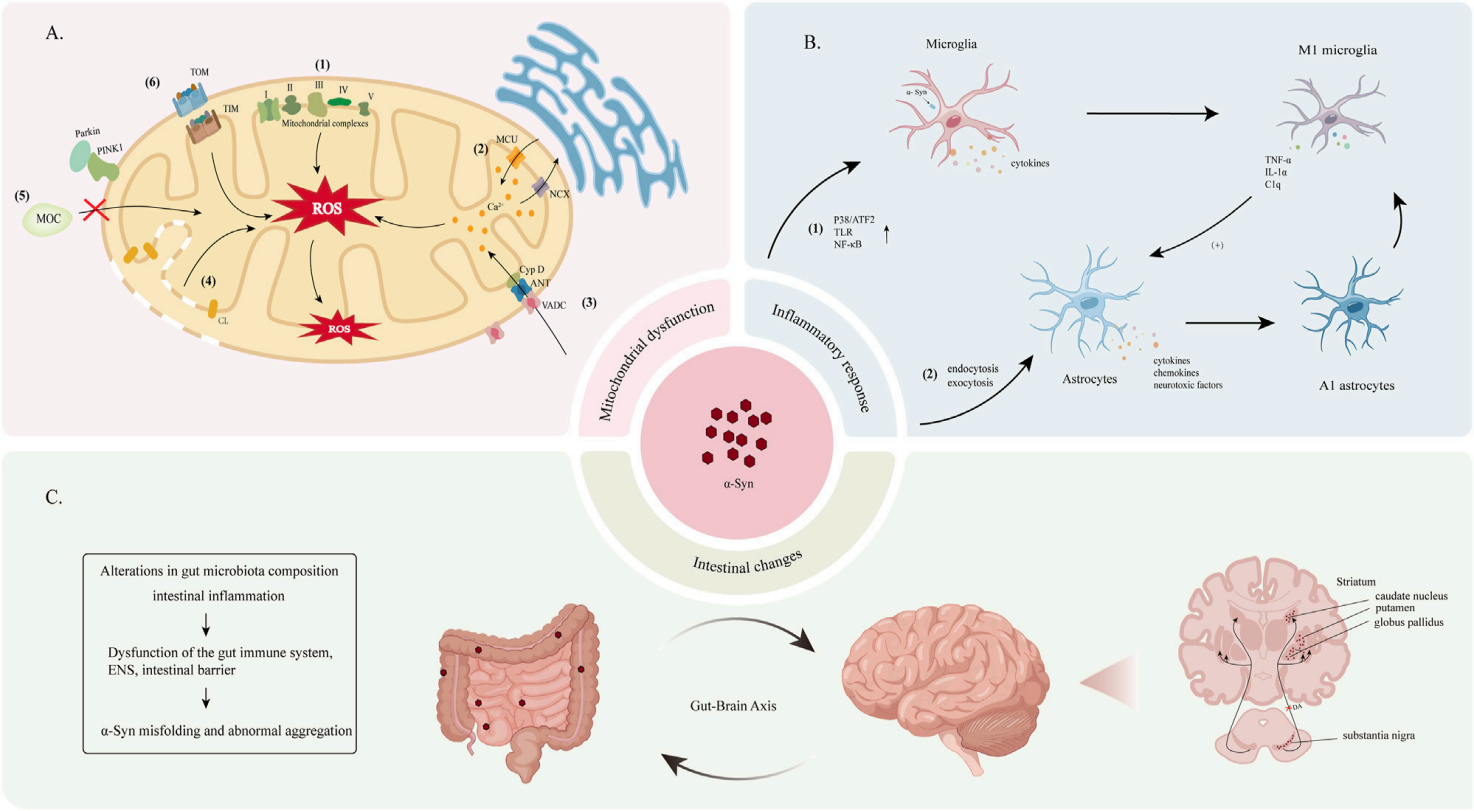
**(A)** The underlying mechanism of α-Syn-induced mitochondrial dysfunction leads to increased intracellular ROS. (1) α-Syn disrupts the activity of mitochondrial electron transport chain complexes, including complex I (Luth et al., 2014), III (Ellis et al., 2005), IV (Danyu et al., 2019; Elkon et al., 2002) and V (Ludtmann et al., 2018), with the most pronounced damage observed in complex I. (2) The accumulation of α-Syn affects the stability of the endoplasmic reticulum-mitochondria association, leading to impaired Ca^2+^ transport and subsequent the disruption of mitochondrial calcium homeostasis (Cali et al., 2012; Paillusson et al., 2017). (3) Fibrillated products of α-Syn interact with components of the mitochondrial permeability transition pore, such as the voltage-dependent anion channel (VDAC), adenine nucleotide translocator (ANT), and mitochondrial matrix protein cyclophilin D (CypD), This interaction activates the mitochondrial permeability transition pore and altering mitochondrial permeability (Torpey et al., 2020; Halestrap, 2009; Lu et al., 2013; Zhu et al., 2011). (4) Cardiolipin (CL)-rich environments enhance the interaction between α-Syn and mitochondria, increasing mitochondrial membrane permeability (Ghio et al., 2019). (5) α-Syn interacts with Parkin and PINK1, disrupting the mitochondrial quality control (MQC) pathway (Thorne and Tumbarello, 2022). (7) α-Syn binds to the TOM and TIM proteins on the outer mitochondrial membrane (OMM) and inner mitochondrial membrane (IMM), interfering with the mitochondrial protein import mechanism (Thorne and Tumbarello, 2022). **(B)** Pathological α-Syn activates microglia and astrocytes, leading to sustained neuroinflammation and neurotoxicity. (1) Pathological α-Syn enhances the activation of p38/ATF2, TLR, and NF-κB in microglia, and promoting their transition to the M1 phenotype. (2) α-Syn enters and activates astrocytes through endocytosis and exocytosis (Lee et al., 2010). Activated astrocytes produce cytokines, chemokines, and toxic factors, which further enhance microglial activation. M1-type microglia release tumor necrosis factor-alpha (TNF-α), interleukin-1α (IL-1α), and complement component 1q (C1q), promoting the formation of A1-type astrocytes. **(C)** Overexpression and abnormal aggregation of α-Syn in the gastrointestinal tract can lead to its transport to the brain via the gut-brain axis, resulting in accumulation within the CNS. α-Syn is predominantly deposited in several brain regions, with the SN and striatum being the most commonly affected areas.

The toxicity of α-Syn to dopaminergic neurons is multifaceted. Mitochondria, the primary source of reactive oxygen species (ROS) within cells, are particularly vulnerable to α-Syn aggregation. This aggregation is believed to induce oxidative stress and mitochondrial dysfunction, both of which are critical factors contributing to the deformation and death of DA neurons in PD. Studies have revealed that α-Syn negatively impacts mitochondria through various mechanisms including binding to mitochondrial respiratory chain complexes, interacting with the mitochondrial permeability transition pore, interfering with mitochondrial protein import, and disrupting mitochondrial quality control (Sohrabi et al., 2023) (Figure 3A). Moreover, α-Syn aggregation disrupts the ALP and UPS, the two major protein degradation pathways in cells, thereby impairing intracellular protein transport and the clearance or damaged proteins (Xilouri et al., 2013a).

Under physiological conditions, microglia and astrocytes function as key central nervous system (CNS) immune and supporting cells, respectively. However, in the presence of pathological α-Syn, microglial proliferation is promoted through mechanisms such as the activation of Toll-like receptors (TLR) and the p38/ATF2 and nuclear factor κB (NF-κB) signaling pathways. This activation leads to the production of various inflammatory factors and ROS, ultimately contributing to DA neurons dysfunction and death (Kam et al., 2020). Furthermore, the accumulation of α-Syn in astrocytes induces the production of proinflammatory factors, chemokines, and neurotoxic factors, further propagating microglial activation (Miyazaki and Asanuma, 2020). (Figure 3B).

One theory proposes that α-Syn aggregates activate microglia to differentiate into a pro-inflammatory phenotype (M1 phenotype). These activated M1 microglia secrete factors such as IL-1α, TNF-α, and C1q, which, in turn, induce astrocytes to transform into a neurotoxic phenotype (A1 phenotype) (Liddelow et al., 2017). Both M1 microglia and A1 astrocytes release inflammatory factors and chemokines that exacerbate neuroinflammation, potentially enhancing the aggregation and spread of α-Syn (Figure 3B). Concurrently, α-Syn activates pericytes, leading to BBB disruption and the infiltration of CD4^+^ and CD8^+^ T lymphocytes, further mediating neuroimmune responses (Delgado-Minjares et al., 2021). In this context, the chronic activation of microglia and astrocytes perpetuates neuroinflammation and neurotoxic responses, contributing to the pathogenesis of PD.

The gut-brain hypothesis posits that the pathological processes of PD may originate in the gut and subsequently spread to the brain, a concept supported by evidence from rat models (Holmqvist et al., 2014). α-Syn may induce alterations in gut microbiota composition and the presence of intestinal inflammation, leading to dysfunction within the gut immune system, enteric nervous system (ENS), and intestinal barrier. Furthermore, the activation of enteric glial cells, increased intestinal permeability, and oxidative stress can elevate α-Syn expression levels in the gut, potentially leading to its misfolding and abnormal aggregation. Moreover, chronic peripheral inflammation may compromise BBB integrity, facilitating the transport of α-Syn from the gut to the CNS (Tan et al., 2022; Houser and Tansey, 2017) (Figure 3C).

## 3 PD models

### 3.1 Neurotoxin-induced models

#### 3.1.1 6-Hydroxydopamine (6-OHDA)

6-OHDA shares structural similarities with DA and norepinephrine, rendering it selectively toxic to catecholaminergic neurons. Due to its inability to cross the BBB, 6-OHDA typically requires direct, targeted injection into the brain to induce neurotoxicity. Once inside the brain, 6-OHDA is taken up by DA/norepinephrine membrane transporters (DMT/NMT), leading to intracellular accumulation and the generation of hydrogen peroxide and ROS. This, in turn, exacerbates oxidative stress, inhibits mitochondrial complex activity, and induces mitochondrial dysfunction, ultimately culminating in neuronal degeneration within specific brain regions. Targeted injection of 6-OHDA into the SN pars compacta, striatum, or medial forebrain bundle disrupts the dopaminergic nigrostriatal system, producing classic Parkinsonian motor deficits in mice. This process effectively recapitulates the progression of pathological and clinical manifestations observed in PD, establishing 6-OHDA as a widely used neurotoxin for inducing PD animal models (Simola et al., 2007).

#### 3.1.2 1-Methyl-4-phenyl-1,2,3,6-tetrahydropyridine (MPTP)

MPTP, due to its high lipophilicity, readily crosses the BBB to reach the CNS. Within astrocytes, MPTP is metabolized to 1-methyl-4-phenylpyridinium (MPP^+^), which then enters dopaminergic neurons via the DA transporter. Inside neurons, MPP^+^ binds to neuromelanin and is transported and stored in synaptic vesicles via the vesicular monoamine transporter type 2 (VMAT-2). Prolonged exposure to MPP^+^ activates microglia, triggering the release of proinflammatory factors that contribute to neurotoxic responses. Furthermore, MPP^+^ inhibits the activity of mitochondrial complex I and suppresses the expression of anti-apoptotic proteins, ultimately impairing ATP synthesis within the electron transport chain and disrupting mitochondrial function. This disruption leads to excessive ROS production, which further promotes the generation and aggregation of α-Syn into toxic oligomers. The cumulative effects of these mechanisms damage dopaminergic neurons in the nigrostriatal pathway, effectively simulating the pathophysiological processes of PD (Mustapha and Taib, 2021).

### 3.2 Pesticide-induced models

Epidemiological studies have established a strong correlation between pesticide exposure and an increased risk of developing PD. Currently, rotenone and paraquat, two widely used pesticides, are frequently employed to generate PD models. Rotenone, a potent mitochondrial complex I inhibitor, shares a mechanism of action like that of MPTP. Due to its high lipophilicity, rotenone readily crosses the BBB, inhibiting mitochondrial complex I function and inducing widespread mitochondrial dysfunction. Additionally, rotenone activates microglia, exacerbating oxidative stress and promoting the accumulation of α-Syn. Research suggests that chronic, low-dose administration of rotenone may more accurately recapitulate the pathophysiology of PD (Innos and Hickey, 2021; Ibarra-Gutiérrez et al., 2023). In contrast, paraquat, an herbicide structurally similar to MPTP, does not inhibit mitochondrial complexes. Instead, paraquat exerts its toxic effects by disrupting the redox cycling of mitochondria, effectively diminishing cellular antioxidant capacity. The toxicity of paraquat to dopaminergic neurons is thought to be mediated by the DMT. Specifically, when paraquat (PQ^2+^) is reduced to the monovalent cation (PQ^+^), it can act as a substrate for DMT, leading to its accumulation within dopaminergic neurons and subsequent oxidative stress and cytotoxicity (Rappold et al., 2011).

### 3.3 Genetic models

Genetic mutations are strongly implicated in familial or genetic forms of PD. Genes such as *SNCA*, *PRKN*, *LRRK2*, and *GBA* are recognized risk factors for PD. Consequently, gene knockout represents a viable approach to model PD in animals. In previous studies, most models lacking specific PD-related genes have exhibited pathophysiological hallmarks of PD, including dopaminergic neurons degeneration, mitochondrial dysfunction, oxidative stress, neuroinflammation, and characteristic motor deficits such as bradykinesia. Furthermore, transgenic animal models have been developed to mimic PD pathology *in vivo* by expressing mutant *SNCA* genes, including those encoding wild-type, A53T, A30P and E46K α-Syn proteins (Lim and Ng, 2009).

## 4 Natural products (NPs)

NPs are compounds derived from natural sources such as plants, animals, and microorganisms. NPs have been extensively utilized in drug development and disease treatment. With advancements in modern chemistry and pharmacological techniques, the isolation and characterization of NPs have become increasingly sophisticated, providing new avenues for drug discovery. In recent years, studies have demonstrated that many NPs possess significant biological activity and exhibit promising therapeutic effects in a wide range of diseases, including cardiovascular diseases, gastrointestinal disorders, respiratory diseases, and infectious diseases (Joo, 2014; Peter et al., 2021). Given that PD remains an incurable neurodegenerative disorder, and considering the limitations and adverse effects associated with existing pharmacological treatments, researchers have turned their attention to exploring NPs as potentially safer and more effective therapeutic or adjunct agents for PD.

### 4.1 Polyphenolic compounds

Polyphenolic compounds, a class of NPs ubiquitous in plants, are primarily obtained from dietary sources such as fruits, vegetables, tea, red wine, and certain nuts. Their characteristic chemical structure, typically comprising multiple hydroxyl and aromatic rings, confers potent antioxidant properties. Polyphenols can be broadly categorized into flavonoids, phenolic acids, anthocyanins, tannins, and other subgroups. These compounds exhibit a wide array of biological activities, including antioxidant, anti-inflammatory, anti-tumor, antibacterial, and cardiovascular protective effects, highlighting their potential therapeutic value in preventing and treating various chronic diseases. Research suggests that a reasonable intake of polyphenolic compounds may contribute to improved health outcomes and a reduced risk of disease development (de Araújo et al., 2021).

#### 4.1.1 Curcumin

Curcumin (Figure 4.1), a natural polyphenolic compound found in turmeric, possesses a distinctive chemical structure of 1,6-heptadiene-3,5-dione-1,7-bis(4-hydroxy-3-methoxyphenyl)-(1E, 6E) (Goel et al., 2008). It exhibits high solubility in organic solvents but poor solubility in aqueous solutions. Additionally, curcumin remains stable in acidic environments but readily decomposes under neutral and alkaline conditions (Wang et al., 1997). Curcumin has long been recognized for its diverse biological activities, including antioxidant (Menon and Sudheer, 2007), anti-inflammatory (Menon and Sudheer, 2007), anticancer (Cheng et al., 2001), antimicrobial (Shao et al., 2024), antithrombotic (Keihanian et al., 2018), blood sugar-lowering (Nabavi et al., 2015), and cardioprotective effects (Zhang et al., 2020).

**FIGURE 4:**
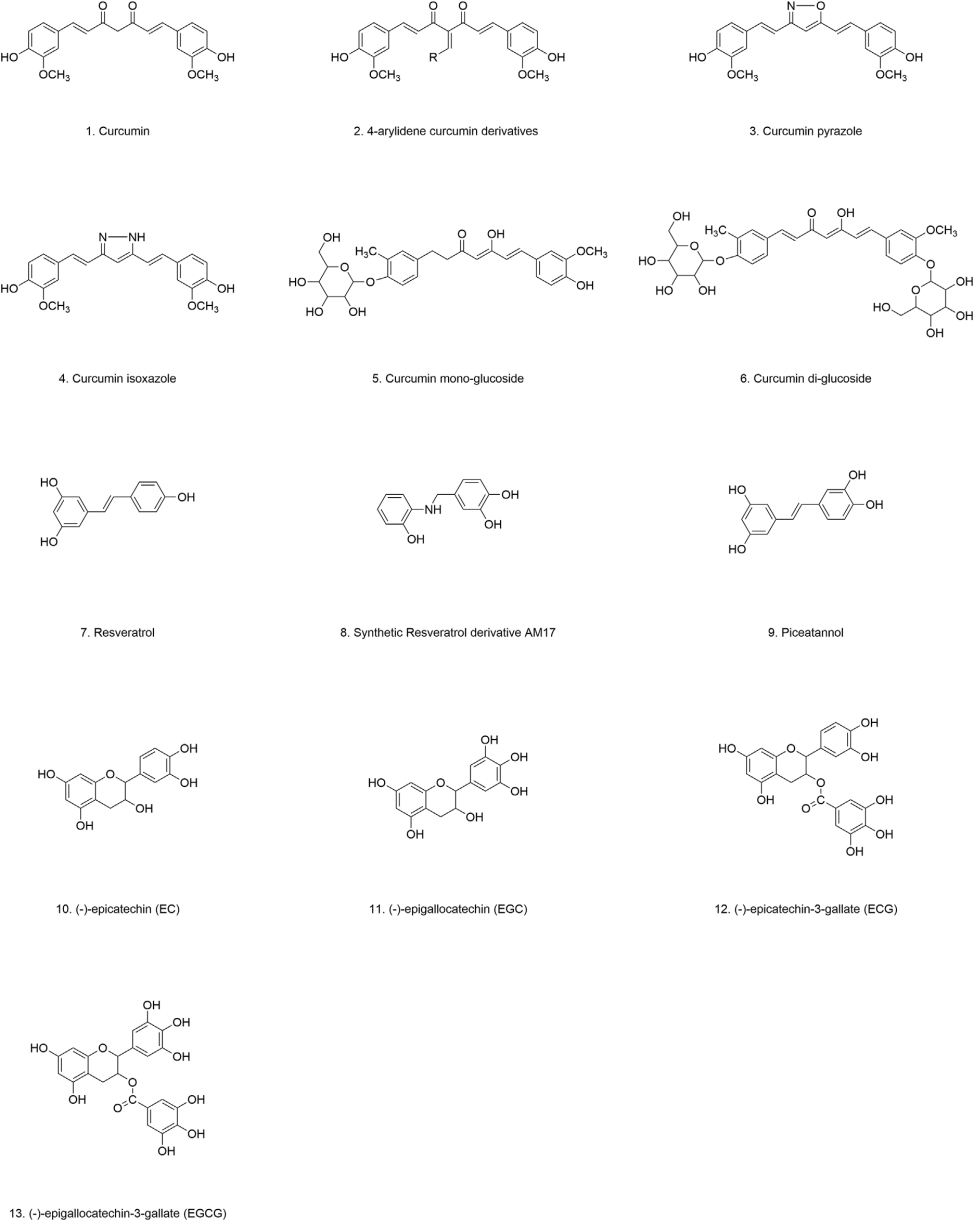
Chemical structure of polyphenolic compounds.

Curcumin’s remarkable ability to penetrate the BBB has led to its widespread investigation as a potential therapeutic agent for neurodegenerative diseases, including PD. Sharma et al. demonstrated in a PD animal model that curcumin treatment significantly inhibited astrocyte activation, downregulated NF-κB transcription factor expression, and suppressed the production of inflammatory factors, ultimately modulating α-Syn aggregation. Moreover, curcumin supplementation was found to inhibit the α-Syn formation and aggregation by suppressing α-Syn gene expression (Sharma and Nehru, 2018).

Several studies suggest that curcumin exerts its inhibitory effects by directly interacting with α-Syn during its phase separation. For instance, Xu et al. reported that while curcumin does not interfere with the initial formation and conformation of α-Syn condensates, it significantly inhibits their transformation into amyloid-like proteins by reducing α-Syn mobility within these condensates (Xu et al., 2022). Similarly, Ahmad et al. discovered that curcumin possesses a unique remodeling capacity. Specifically, its interaction with α-Syn monomers disrupts long-range interactions within the protein chain, leading to an increased refolding rate, prevention of α-Syn aggregation, and temperature-dependent reaction kinetics (Ahmad and Lapidus, 2012).

The dose-dependent inhibitory effect of curcumin on α-Syn aggregation was further corroborated by Pandey et al., who observed that curcumin, particularly at concentrations of 10^−6^–10^−7^ M, significantly increased the soluble fractions of α-Syn monomers, dimers, and oligomers. This increase in solubility reduces the stability of these aggregates, rendering them more susceptible to degradation (Pandey et al., 2008). These findings align with those of Ono et al., who also emphasized the importance of curcumin dosing in inhibiting α-Syn aggregation and fibril growth (Ono and Yamada, 2006). Furthermore, curcumin has been shown to bind to preformed α-Syn fibrils and aggregates, altering their hydrophobic surface structure and reducing α-Syn toxicity. Intriguingly, curcumin appears to selectively bind to the ordered structure of the protein, with the degree of binding correlating with the extent of α-Syn oligomerization (Singh D. et al., 2012).

Research on curcumin as an adjunct therapy for cancer has been extensive, consistently demonstrating its favorable safety profile. Curcumin is generally well-tolerated, even at single oral doses as high as 12 g/d. However, its clinical application and development have been hampered by its poor solubility, inherent instability, and suboptimal pharmacokinetic profile. Specifically, inadequate intestinal absorption, rapid metabolism, and rapid systematic elimination result in low plasma and tissue concentrations, limiting its therapeutic efficacy. Interestingly, co-administration with piperine, a natural alkaloid found in black pepper, has been shown to increase curcumin bioavailability by an impressive 2,000%, highlighting the potential of piperine as a bioenhancer for curcumin (Sharma et al., 2007; Anand et al., 2007).

To address the inherent limitations of curcumin, researchers have focused on synthesizing curcumin analogs with enhanced biological activity. For instance, Jha et al. synthesized a series of curcumin analogs by substituting the hydroxyl group on the phenyl ring with various functional groups. These modifications differentially reduced the hydrophobic surface exposure of α-Syn oligomers, effectively mitigating their cytotoxicity (Jha et al., 2016). Notably, 4-arylidene curcumin derivatives (Figure 4.2) exhibited superior anti-aggregation properties, effectively targeting both α-Syn fibrils and oligomers (Liu W. et al., 2023). Similarly, two other stable curcumin analogs, curcumin pyrazole (Figure 4.3) and curcumin isoxazole (Figure 4.4), have demonstrated promising anti-amyloidogenic activity, effectively preventing fibrillation, disrupting preformed fibrils, and inhibiting the formation of toxic A11 protein conformations (Ahsan et al., 2015). Furthermore, Gadad et al. synthesized curcumin-glucoside (Figures 4.5, 4.6), which also exhibited robust inhibitory effects on α-Syn aggregation and fibril formation under *in vitro* conditions (Gadad et al., 2012). These findings collectively suggest that curcumin and its related polyphenolic compounds hold significant promise as therapeutic candidates for PD and other related neurodegenerative disorders (Table 1).

**TABLE 1:** Positive effects of polyphenolic compounds targeting α-Syn *in vivo* and *in vitro* models of PD.

Natural products	*In vitro* and *in vivo* model	Effects and mechanisms observed	Reference
Curcumin	Lipolyaccharide-induced PD model	1.Induces astrocytic activation, downregulates the expression of NF-κB and inflammatory factors 2.Prevents the expression of the α-Syn gene, inhibits the formation and aggregation of α-Syn	Sharma and Nehru (2018)
	α-Syn aggregation assay	1.α-Syn undergoes phase separation to accelerate amyloid aggregation, decreases the fluidity of α-Syn 2.Destabilizes preformed α-Syn amyloid aggregates	Xu et al. (2022)
	α-Syn aggregation assay SH-SY5Y cells expressing A53T-α-Syn	1.Binds to α-Syn monomers, prevents aggregation, and increases the reconfiguration rate 2.Increases the solubility of the α-Syn monomers, decreases aggregation	Ono and Yamada (2006), Ahmad and Lapidus (2012), Pandey et al. (2008)
	α-Syn aggregation assay	1.Binds to preformed oligomers and fibrils and alters hydrophobic surface exposure, reducing toxicity 2.Alters morphology of the α-Syn oligomers, reduces the exposed hydrophobic surface of α-Syn oligomers, inhibits α-Syn aggregation	Singh P. K. et al. (2012)
Curcumin analogs	α-Syn aggregation assay SH-SY5Y cells	1.Structurally similar to curcumin, but have different functional groups and hydrophobicity, is more stable than curcumin 2.Interacts with preformed fibrils and oligomers and accelerates α-Syn aggregation to produce morphologically different amyloid fibrils and reduce toxicity	Jha et al. (2016)
4-arylidene curcumin derivatives	α-Syn aggregation assay	1.Inhibits α-Syn aggregation and fibrils formation, stabilizes α-Syn structure and prevents β-sheet aggregation 2.Disintegrates preformed α-Syn oligomers and fibrils 3.Shows stronger inhibition and disintegration activity	Liu W. et al. (2023)
Curcumin pyrazole and curcumin isoxazole	α-Syn aggregation assay SH-SY5Y cells	1.Inhibits α-Syn aggregation, fibrillization and toxicity 2.Prevents formation of A11 conformation in the protein	Ahsan et al. (2015)
Curcumin-glucoside	α-Syn aggregation assay	1.Prevents oligomers formation and inhibits fibrils formation 2.Solubilizes the oligomeric form by disintegrating preformed fibrils	Gadad et al. (2012)
Resveratrol	Modeling experiments utilizing hydrogen/deuterium exchange mass spectrometry	1.α-Syn misfolding is most evident within and nearby the NAC region 2.Significantly remodels α-Syn aggregation	Illes-Toth et al. (2024)
	MPTP-induced mice model	1.Activates SIRT1, promotes α-Syn autophagy degradation,and reduces the accumulation of α-Syn 2.Improves the behavioral impairments of MPTP-treated mice 3.Attenuates MPTP-induced depletion of DA in the striatum 4.Salvages the loss of nigra neurons and striatal proteins	Guo S. S. et al. (2016)
Synthetic resveratrol derivative AM17	α-Syn aggregation assay	1.Inhibits α-Syn monomers aggregation 2.Disaggregates α-Syn oligomers and fibrils independent of copper ions	Chau et al. (2021)
Piceatannol	α-Syn aggregation assay PC12 cells	1.Inhibits the formation of α synuclein fibrils and destabilizes preformed filament 2.Induces the formation of small soluble complexes protecting membranes against α-Syn-induced damage 3.Protectes cells against α-Syn-induced toxicity	Temsamani et al. (2016)
(−)-epigallocatechin-3-gallate (EGCG)	α-Syn aggregation assay	Binds to cross-β sheet aggregation and mediates the conformational change, reduces toxicity	Andrich and Bieschke (2015)
	*In vitro* membrane permeabilization assay Immortalized oligodendroglial cell line OLN-93	1.Immobilizes the C-terminal region and reduces the binding of oligomers to membranes 2.Inhibits preformed oligomers permeabilize vesicles 3. Oligomers destabilize the membrane	Lorenzen et al. (2014)
	α-Syn aggregation assay	1.Dose-dependently inhibits α-Syn aggregation 2.Reduces the α-Syn oligomers/fibers volume 3.Reduces hydrophobic surface exposure and the toxicity of α-Syn	Jha et al. (2017)
	Microsecond all-atom molecular dynamics simulations test	Forms H-bonding and cation-π interactions with membrane, attenuates protofibrils-membrane interactions, impedes the membrane damage by α-Syn protofibrils and enables the membrane integrity	Yang B. et al. (2023)
	α-Syn aggregation combined analysis detection α-Syn transduced PC12 cells	1.Binding to Ile, Phe and Tyr amino residues to inhibit α-Syn conformational transformation 2.Binding to Leu, His, Phe and Tyr amino residues to disaggregate the α-Syn amyloid fibrils 3.Inhibits the overexpression and fibrillation of α-Syn in the cells, reduces the damage to cells	Zhao J. et al. (2017)
	IM-MS	1.Binds randomly to the backbone groups of the α-Syn 2.Protects against the conformational collapse of α-Syn associated with aggregation, inhibiting fibrilization 3.The combination with EGCG results in α-Syn structure rearrangement and stabilizes extended structures	Liu et al. (2011)
	Molecular dynamics simulations test	1.Breaks β-sheets and the global structure of α-Syn fibrils, inhibiting the growth of fibrils 2.Disrupts the E46-K80 Salt-Bridges, reducing the Greek-Key-Like Structure and the Hydrophobic Interactions, reducing the stability of fibrils structure	Yao et al. (2020)
	α-Syn aggregation assay Quantitative seeding experiments	1.The oxidation products of EGCG show a stronger inhibitory effect, inducing primary nucleation 2.EGCG and oxidation products act as enhancer of amyloid fibrils formation under certain conditions	Sternke-Hoffmann et al. (2020)

#### 4.1.2 Resveratrol

Resveratrol (Figure 4.7), a low molecular-weight polyphenolic compound, is abundant in various plants and trees, including pine, eucalyptus, grapes, blueberries, and cranberries. It exhibits a wide range of protective activities, including antioxidant, anti-inflammatory, free radical scavenging, and neuroprotective effects, establishing its potential as a dietary supplement for neurodegenerative diseases (Bastianetto et al., 2015). Kinetic modeling experiments employing hydrogen/deuterium exchange mass spectrometry have revealed that resveratrol significantly remodels α-Syn aggregation (Illes-Toth et al., 2024).

In PD models induced by 6-OHDA, MPTP, and rotenone, resveratrol effectively ameliorates motor deficits in mice (Zhao X. et al., 2017; Jin et al., 2008; Lu et al., 2008; Liu D. et al., 2019). It reduces α-Syn expression and toxicity while enhancing cell survival, likely through the activation of sirtuin 1, which promotes α-Syn degradation via autophagy (Guo S. S. et al., 2016). Resveratrol analogs have also demonstrated comparable neuroprotective effects. For instance, the resveratrol derivative AM17 (Figure 4.8) and piceatannol (Figure 4.9) both inhibit α-Syn monomer aggregation and disassemble preformed α-Syn oligomers and fibrils, independent of copper ions (Temsamani et al., 2016; Chau et al., 2021).

Although resveratrol readily crosses the BBB (Marier et al., 2002), its rapid metabolism, low bioavailability, and inherent chemical instability limit its clinical utility, even at high concentrations (Calamini et al., 2010). While resveratrol exhibits an oral absorption rate of approximately 75%, it undergoes rapid first-pass metabolism and elimination, resulting in low systemic exposure. Furthermore, circadian rhythms may influence resveratrol bioavailability. Studies suggest that morning administration may enhance its absorption. The recommended oral dosage range for resveratrol is 100–1,000 mg. Doses exceeding 2 g/d may lead to adverse effects such as diarrhea, nausea, vomiting, or headaches (Vesely et al., 2021).

To overcome these limitations, structural modifications aimed at improving bioavailability, metabolic stability, and biological activity without compromising its protective effects are being actively pursued. Current modification strategies include hydroxylation, amination/amidation/imination, methoxylation, prenylation, and glycosylation. Notably, resveratrol derivatives and analogs, such as oxyresveratrol, piceatannol, and imine resveratrol derivatives, exhibit more favorable pharmacokinetic profiles compared to the parent compound, demonstrating faster absorption, higher bioavailability, and greater metabolic stability (Li S. Y. et al., 2014; Setoguchi et al., 2014; Jung et al., 2009). Moreover, novel drug delivery systems, such as nanoparticle-loaded resveratrol and vitamin E-loaded resveratrol nanoemulsions, have shown reduce oxidative stress, enhance intracranial drug concentrations, and diminish degenerative lesions, demonstrating promising therapeutic effects against PD (Pangeni et al., 2014) (Table 1).

#### 4.1.3 Green tea catechins

The major polyphenolic compounds found in green tea are collectively known as catechins (flavan-3-ols). Catechins have demonstrated a wide array of beneficial health effects, including anti-inflammatory, antioxidant, antibacterial, anticancer, infection-preventive, cognitive-enhancing, memory-improving, and neuroprotective properties. These compounds have been implicated in the treatment of chronic diseases such as cardiovascular diseases, diabetes, obesity, and neurodegenerative disorders. Green tea catechins primarily include (-)-epicatechin (EC) (Figure 4.10), (-)-epigallocatechin (EGC) (Figure 4.11), (-)-epicatechin-3-gallate (ECG) (Figure 4.12), and (-)-epigallocatechin-3-gallate (EGCG) (Figure 4.13). The antioxidant activity of these catechins varies depending on the number of hydroxyl groups and the structure of their substituent groups. EGCG is the most abundant catechin in green tea, followed by EGC (Musial et al., 2020).

Studies have shown that green tea catechins can mitigate α-Syn pathology in PD models. For instance, in MPTP-induced PD monkeys, administration of catechin-rich tea polyphenol extracts significantly reduced α-Syn aggregation in the striatum and hippocampus, attenuating α-Syn-induced dopaminergic neurons loss and motor dysfunction (Chen et al., 2015). Using parallel mass spectrometry to analyze green tea metabolites, Williams et al. found that, in addition to EGCG, both catechin and EC not only inhibited the formation of α-Syn fibrils but also destabilized preformed α-Syn fibrils (Williams et al., 2007).

To date, EGCG (Figure 4.13) is one of the most extensively studied and bioactive catechins extracted from green tea. In preformed α-Syn fibril models, EGCG disrupts the structural integrity of α-Syn fibrils by interfering with hydrogen bonds, aromatic stacking, and cation-π interactions, thereby inhibiting α-Syn fibrillation. In cellular models, EGCG directly binds to the β-sheet structure of α-Syn aggregates, inducing conformational changes that inhibit aggregation and promote the breakdown of aggregates into smaller, less toxic, and more disordered species (Andrich and Bieschke, 2015; Bieschke et al., 2010; Dominguez-Meijide et al., 2020). The destabilizing effect of EGCG on α-Syn fibrils is closely associated with the disruption of the E46-K80 salt bridge (Yao et al., 2020). Moreover, EGCG interaction with α-Syn reduces the affinity between α-Syn and lipid membranes, thereby mitigating hydrophobic surface exposure and cytotoxicity (Zhao J. et al., 2017; Lorenzen et al., 2014; Jha et al., 2017; Andrich and Bieschke, 2015; Yang Z. et al., 2023).

Furthermore, EGCG has been shown to interfere with copper (II) (Cu(II))-induced ROS generation, protecting cells from the toxic effects of α-Syn overexpression and fibrillation (Teng et al., 2019). Additionally, EGCG may influence α-Syn expression *in vivo* by modulating both the expression of the *SNCA* gene and the methylation status of CpG sites within its promoter region (Ramakrishna et al., 2016). In MPTP-treated mice, EGCG attenuates α-Syn accumulation and reduces neuronal death, potentially through mechanisms involving increased Bcl-2 protein expression, suppressed Bax protein expression, and upregulation of the PKC pathway (Mandel et al., 2004).

Ion mobility-mass spectrometry (IM-MS) confirmed that EGCG binds to α-Syn in a non-specific manner, inducing a more compact protein structure that resists conformational changes and reduces fibrillar aggregate formation (Liu et al., 2011). Interestingly, EGCG oxidation products exhibit even stronger inhibitory effects on α-Syn aggregation than the parent compound. Paradoxically, some studies have reported that both EGCG and its oxidation products can accelerate α-Syn fibril formation. This discrepancy may be attributed to variations in experimental conditions and warrants further investigation (Sternke-Hoffmann et al., 2020) (Table 1).

Despite its potent neuroprotective properties, the therapeutic application of EGCG is hindered by its inherent instability and poor bioavailability. The rapid metabolic degradation of EGCG often necessitates high doses to achieve therapeutic efficacy. However, such high doses may lead to toxicity, presenting a significant challenge for clinical transition. To overcome these limitations, researchers are actively exploring strategies to enhance EGCG bioavailability and reduce its toxicity, including chemical modification and nanoparticle-based delivery systems (Mehmood et al., 2022).

#### 4.1.4 Flavonoid-like compounds

Flavonoids, the most abundant and diverse group of polyphenolic compounds, are found ubiquitously in plants and dietary sources. They share a common structural backbone consisting of a 2-phenyl-benzo-alpha-pyran or flavan nucleus, comprising two benzene rings (A and B) linked by a C heterocyclic pyran ring. Based on variations in the pyran ring structure, flavonoids are classified into six major subclasses: flavonols, flavanones, flavanols, flavones, anthocyanins, and isoflavones. Recognized for their wide-ranging biological activities, flavonoids are often consumed as dietary supplements, offering numerous health benefits. The recommended daily dosage for flavonoids typically ranges from 500 to 1,000 mg (Billowria et al., 2024).

##### 4.1.4.1 Hesperetin and hesperidin

Hesperetin (HST) (Figure 5.1) is a naturally occurring bioflavonoid found abundantly in citrus fruits (*Rutaceae*), including lemons, oranges, limes, tangerines, and grapefruits. Both HST and its glycoside derivative, hesperidin (HSD) (Figure 5.2), are potent antioxidants that effectively protect cells from oxidative stress-induced damage. Their anti-oxidant, anti-inflammatory, and neuroprotective properties have been implicated in mitigating the progression of neurodegenerative diseases, including PD (Evans et al., 2022; Cho, 2006; Hajialuani et al., 2019). Moreover, HST and HSD have demonstrated efficacy in enhancing both non-spatial and spatial learning and memory. These beneficial effects are attributed to their ability to bolster antioxidant defenses and enhance cholinergic and brain-derived neurotrophic factor (BDNF) signaling. Furthermore, HST and HSD have been shown to reduce dopaminergic neurons injury, mitochondrial dysfunction, and apoptosis in PD mouse models (Antunes et al., 2021; Ishola et al., 2019). Importantly, HSD, when administered in conjunction with low-dose levodopa, significantly inhibits ROS accumulation and oxidative stress-induced damage while ameliorating mitochondrial dysfunction and motor deficits (Kesh et al., 2021). This finding suggests that combining HSD with conventional PD medications may synergistically enhance therapeutic outcomes.

**FIGURE 5:**
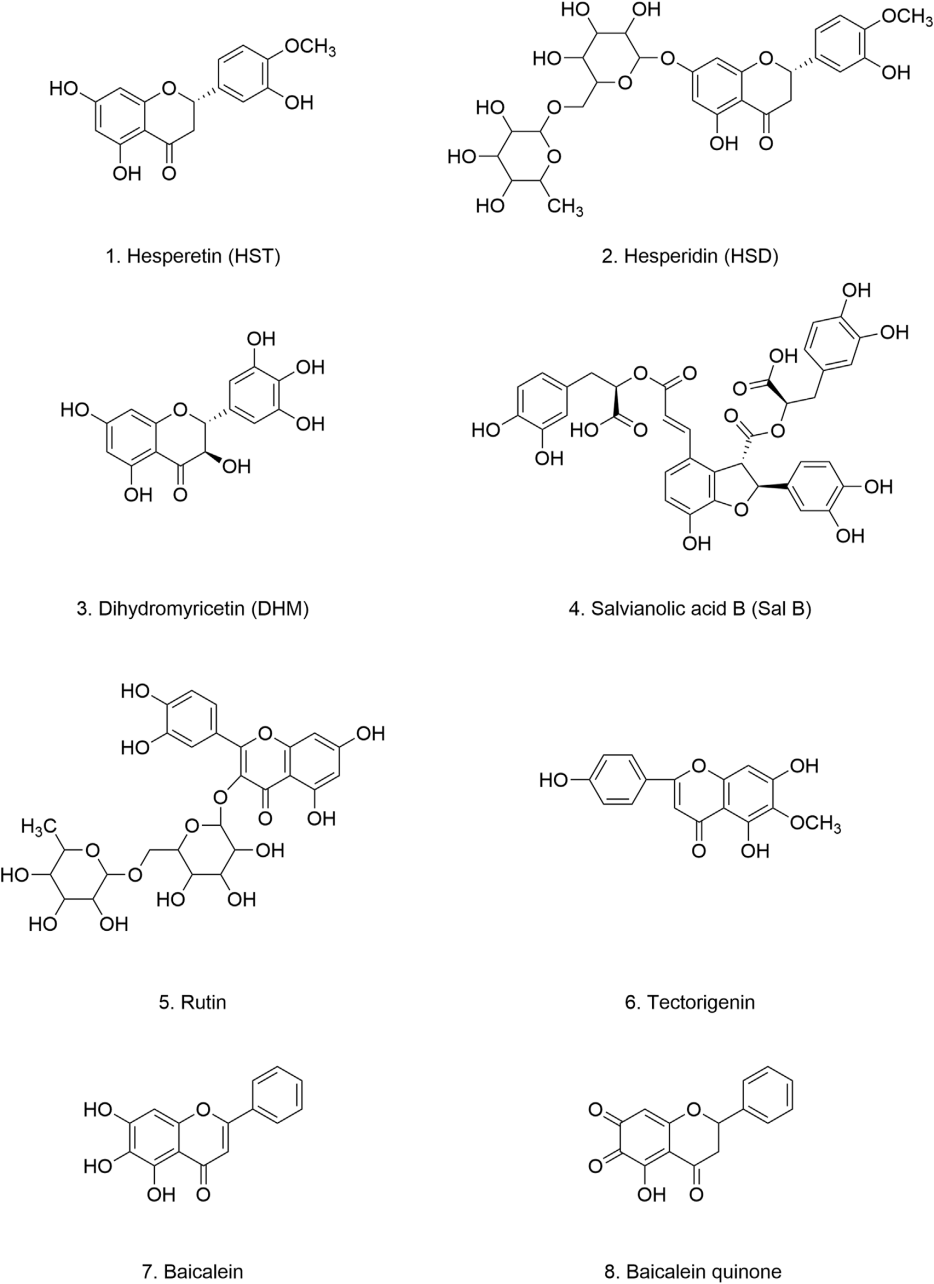
Chemical structure of flavonoid-like compounds.

In cellular experiments, Wang et al. demonstrated that HST effectively inhibits α-Syn fibril formation by interfering with both the initial nucleation and elongation phases. Moreover, HST interacts with α-Syn monomers and disassembles preformed α-Syn aggregates, leading to the formation of shorter, thinner, and less structured oligomers. This structural remodeling reduces the cytotoxicity and neurotoxicity associated with α-Syn aggregates.

The protective effects of HST have been observed in *in vivo* models as well. In the Transgenic *Caenorhabditis elegans* (*C. elegans*) model, HST treatment significantly reduced α-Syn aggregation in the NL5901 strain, extending lifespan and improving overall health (Wang Q. et al., 2023). In *Drosophila* models, HST demonstrated beneficial effects on reproductive capacity and locomotor activity, suggesting its potential in ameliorating PD-related motor dysfunction (Ishola et al., 2021). HSD also exerts protective effects against α-Syn pathology. It downregulates the expression of key kinases involved in α-Syn production, including LRRK2, GSK3β, caspase-3, caspase-9, and POLG, thereby reducing α-Syn levels and cytotoxicity (Kesh et al., 2021). Notably, the effects of HST are concentration-dependent, with a cell viability rate of up to 98.4% observed at a concentration of 100 μM HST, indicating a favorable safety and tolerability profile (Wang W. et al., 2023) (Table 2).

**TABLE 2:** Positive effects of flavonoid-like compounds targeting α-Syn *in vivo* and *in vitro* models of PD.

Natural products	*In vitro* and *in vivo* model	Effects and mechanisms observed	Reference
Hesperidin	6-OHDA treated SH-SY5Y cells and zebrafish model	1.Alleviates 6-OHDA-induced toxicity and oxidative stress 2.Rescues Mitochondrial membrane potential 3.Improves locomotor behavior 4.Downregulates the expression of *LRRK2*, *polg*, *GSK3β*, and *casp9*	Kesh et al. (2021)
Hesperetin	α-Syn aggregation assay α-Syn treated PC12 cells and *C.elegans* NL5901	1.Interferes with α-Syn initial nucleation and slows the elongation rate, inhibits α-Syn fibrils formation 2.Decomposes α-Syn preformed aggregates and reduces their cytotoxicity 3.Reduces α-Syn aggregates of NL5901, restores lipid deposition levels in the nematode and promotes the health and longevity of NL5901	Wang W.et al. (2023)
Dihydromyricetin (DHM) and Salvianolic acid B (Sal B)	α-Syn transfected H4 cells Homozygous transgenic mice expressing WT-α-Syn	1.CMA modulates α-Syn aggregation and toxicity *in vitro* 2.Activate CMA and degrade α-Syn aggregates 3.Decreased astrogliosis and microgliosis, inhibiting neuroinflammation	Wu et al. (2019)
	α-Syn aggregation assay Primary neuronal cells	1.Inhibits α-Syn fibrils formation 2.Stabilizes α-Syn oligomerization 3.Inhibits α-Syn induced cytotoxicity 4.Disaggregates preformed α-Syn amyloid fibrils 5.Interferes with the seeding of α-Syn monomers 6.Affects the seeding dependent aggregation of α-Syn in cells	Ardah et al. (2020)
	α-Syn aggregation assay PC12 neuronal cell line	1.Inhibits α-Syn fibrillogenesis 2.Alters the ultrastructure of α-Syn aggregates 3.Againsts α-Syn fibrils-induced cytotoxicity 4.Disrupts preformed α-Syn fibrils and reduces the neurotoxicity	Jia et al. (2019)
Rutin	A53T α-Syn transfected SH-SY5Y cells	1.Avoids the formation of oxidized lipids during α-Syn challenge 2.Improves neurite outgrowth 3.Protects synaptic vesicles and neurons against α-Syn toxicity	Christmann et al. (2022)
Tectorigenin	α-Syn and tectorigenin aggregation model	1.Binds to α-Syn leads to microenvironmental changes in the tertiary structure of α-Syn 2.Improves thermal stability and chaperon-like activity, leading to stabilize α-Syn	Tu et al. (2023)
Baicalein	α-Syn aggregation assay	1.Combining with α-Syn to form the Schiff Base, inhibit α-Syn fibrils formation 2.Stabilizes a partially folded conformation of α-Syn and oligomers 3.The quinone oxidized form binds α-Syn, stabilizing oligomers to prevent fibrillation 4.Disaggregates existing α-Syn fibrils, form the soluble oligomers again	(Zhu et al., 2004)
	Expression of E46K mutant α-Syn induced PC12 cell lines E46K-transfected N2A cells	1.Inhibits α-Syn aggregation and fibrils formation, prevents α-Syn conversion to β-sheet conformation 2.Changes oligomers structure and α-Syn-induced cytotoxicity	Jiang et al. (2010)
	SH-SY5Y cells, Hela cells and SN4741 cells	1.Decreases α-Syn expression in transfected cells, upregulates the cell viability, enhances macro-autophagy accelerates α-Syn clearance 2.Alleviates the neurotoxicity of α-Syn oligomers	Li et al. (2017a), Lu et al. (2011)
	α-Syn aggregation assay and correlation properties analysis of baicalein and α-Syn conjugate	Induces α-Syn to form stable oligomers, improves thermal stability and reduces the damage to the integrity of LM, inhibits the fibrillation of α-Syn	Hong D. P. et al. (2008)
	Microsecond molecular dynamics simulations WT-α-Syn fibrils, familial PD-associated mutant E46K fibrils, H50Q fibrils	Disrupts E46-K80 salt-bridge and β-sheets transforms them into disordered conformations, remodeling the inter-protofilament interface	Yao et al. (2022)
	Rotenone-induced mouse model	1.Ameliorates the behavioral dysfunction of mice 2.Restores striatal neurotransmitters of mice 3.Prevents α-Syn accumulation 4.Inhibits α-Syn oligomers *in vivo* and *in vitro*	Hu et al. (2016)
	MPP^+^-induced mouse model	1.Prevents MPP^+^-induced neurotoxicity and α-Syn aggregation 2.Prevents MPP^+^-induced inflammasome activation and autophagy apoptosis and autophagy	Hung et al. (2016)
Nanoparticle-modified Baicalein	α-Syn aggregation assay SH-SY5Y and OLN-93 cells	1.Increases drug stability, solubility, and availability 2.Affects α-Syn fibrillation and secondary fibrillation, reduces permeabilization of membranes and cytotoxicity triggered by α-Syn	Aliakbari et al. (2021)

Studies investigating the pharmacokinetics of HST and HSD have reported no significant adverse effects following oral administration. HST is rapidly absorbed into the bloodstream, appearing in plasma within 20 min of ingestion and reaching peak concentrations at 4 h. The serum half-life of HST ranges from 3.7 to 7 h, while its elimination half-life in urine is approximately 25 h (Kanaze et al., 2007; Yáñez et al., 2008). Collectively, these findings suggest that HST, alone or in combination with HSD, holds promise as a dietary supplement for modulating α-Syn fibrillation and aggregation, potentially preventing or delaying the onset and progression of PD.

##### 4.1.4.2 Dihydromyricetin

Dihydromyricetin (DHM) (Figure 5.3) is a bioactive flavonoid found predominantly in the stems and leaves of rattan grapes (*Ampelopsis grossedentata*), where its concentration can reach 30%–40% (Sun et al., 2021). DHM is also present in other medicinal plants and plant-based foods, including grapes, bayberries, *Ginkgo biloba*, *Hovenia dulcis*, and *Cedrus deodara* (Liu M. et al., 2020; Liu Q. et al., 2019). DHM possesses a wide range of pharmacological properties, including antioxidant, free radical scavenging, anti-inflammatory, antitumor, antimicrobial, cell death-modulating, and lipid- and glucose-regulating activities (Li H. et al., 2017). Importantly, acute and long-term toxicity studies, as well as genotoxicity tests, have confirmed the non-toxic nature and long-term safety of DHM (Zhang et al., 2021).

Previous studies have established that chaperone-mediated autophagy (CMA) plays a crucial role in the degradation of α-Syn (Xilouri et al., 2013b; Cuervo et al., 2004). CMA is a highly regulated cellular process that selectively targets proteins for lysosomal degradation (Kaushik and Cuervo, 2008). DHM has been shown to induce autophagy through the regulation of the AMPK/PCG pathway and other signaling pathways (Shi et al., 2015a; Shi et al., 2015b). Building upon these findings, Wu et al. demonstrated that treatment with DHM and salvianolic acid B (Sal B) (Figure 5.4) effectively inhibited the accumulation and aggregation of α-Syn fibrils both *in vitro* and *in vivo*. DHM and Sal B treatment led to a significant increase in the expression of LAMP-2A, a key marker of CMA, confirming the involvement of DHM in this pathway. The authors further showed that DHM and Sal B reduce α-Syn levels by enhancing CMA activation, thereby mitigating cytotoxicity and inhibiting inflammatory responses (Wu et al., 2019). Ardah et al. extended these findings, reporting that both compounds stabilized α-Syn oligomers. Interestingly, they observed distinct binding preferences for each compound. DHM preferentially bound to α-Syn oligomers, while Sal B exhibited higher affinity for α-Syn monomers. Remarkably, DHM also demonstrated the ability to degrade preformed α-Syn fibrils (Ardah et al., 2020). These findings are consistent with those reported by other research groups (Jia et al., 2019) (Table 2).

##### 4.1.4.3 Rutin

Rutin (Figure 5.5) is a widely distributed polyphenolic flavonoid found in various plants and fruits, including buckwheat, apricots, oranges, cherries, and grapes (Huang et al., 2012; Kreft et al., 1999; Ganeshpurkar and Saluja, 2017). Structurally, rutin is a glycoside composed on the flavonol aglycone quercetin and the disaccharide rutinose. Extensive research has highlighted the numerous health-promoting properties of rutin, including antioxidant, neuroprotective, vasoprotective, and cytoprotective effects (Kim et al., 2009; Javed et al., 2012; Enogieru et al., 2018; Kalgaonkar et al., 2010; Kamalakkannan and Prince, 2006; Khan et al., 2009).

The gut-brain axis hypothesis highlights the critical importance of early intestinal intervention in the initial stages of neurodegenerative diseases like PD. Christmann et al. investigated the efficacy of early intervention with rutin supplementation in both ENS and CNS. Their findings demonstrated that rutin effectively mitigated the deleterious effects of α-Syn on cells, promoting neuronal growth and protecting synaptic vesicles and neurons from α-Syn toxicity. These protective effects are attributed to rutin’s ability to reduce lipid peroxidation and scavenge ROS.

Despite its promising preclinical profile, the clinical application of rutin is limited by its poor solubility and absorption, resulting in low oral bioavailability (Liu Y. et al., 2020). Interestingly, Christmann et al. observed that rutin nanocrystals exhibited enhanced protective capabilities compared to conventional rutin particles, suggesting that nanoformulations may improve its bioavailability and therapeutic efficacy (Christmann et al., 2022) (Table 2). These findings underscore the potential of antioxidant dietary supplements like rutin, particularly when administered via the intestinal route, for early prevention and treatment of PD. Further research is warranted to optimize rutin delivery and evaluate its clinical efficacy in PD patients.

##### 4.1.4.4 Tectorigenin

Tectorigenin (Figure 5.6) is a naturally occurring methoxylated flavone found predominantly in the rhizomes of Iridaceous plants, such as *Iris spuria* and *Iris tectorum*. It is also present in other genera, including *Pueraria*, *Morus alba*, and *Codonopsis pilosula* (Rong et al., 2023). Tectorigenin exhibits a wide range of biological activities, including antioxidant, anti-inflammatory, antibacterial, anticancer, hypoglycemic, and hepatoprotective effects (Hong D. P. et al., 2008; Thelen et al., 2005; Jiang et al., 2012; Bae et al., 1999; Lee et al., 2003; Pan et al., 2008; Park et al., 2002). In an MPP^+^-induced cellular model of PD, tectorigenin exhibited neuroprotective effects, potentially by mitigating oxidative stress. Similar to other polyphenolic compounds, tectorigenin readily crosses the BBB, particularly when conjugated to a delivery vehicle. This characteristic makes it a promising candidate for PD therapeutics (Youdim et al., 2004; Gong et al., 2017).

Recent studies have revealed that tectorigenin binds to α-Syn through a combination of hydrogen bonds and van der Waals forces. This interaction induces conformational changes in the tertiary structure of α-Syn, leading to the formation of a stable tectorigenin-α-Syn complex (Tu et al., 2023). Notably, complex formation significantly enhances the thermal stability and chaperone activity of α-Syn. Furthermore, tectorigenin exhibits a higher binding affinity for α-Syn fibrils than for monomers (Table 2).

While generally safe within therapeutic dosage ranges, tectorigenin’s toxicity appears to be dose-dependent. Exceeding a specific concentration or prolonging treatment duration may lead to adverse effects. However, like many flavonoids, its poor water solubility limits its bioavailability. Despite this limitation, tectorigenin’s relatively long half-life suggests a prolonged duration of action *in vivo*. This prolonged activity may offer advantages over other NPs (Rong et al., 2023). However, further research is necessary to confirm these findings. Current knowledge regarding the relationship between tectorigenin and α-Syn aggregation mechanisms in PD remains limited, lacking robust experimental and clinical evidence.

##### 4.1.4.5 Baicalein

Baicalein (Figure 5.7) is a flavonoid compound derived from the rhizomes of *Scutellaria baicalensis*. In addition to the shared biological activities of flavonoids, baicalein has been identified as a potent inhibitor of α-Syn aggregation. Interestingly, its oxidized form demonstrates inhibitory effects even at low concentrations, likely due to its quinone structure (Figure 5.8), which may facilitate interactions with α-Syn and promote the formation of structurally stable, soluble aggregates (Zhu et al., 2004). Baicalein also shows protective effects against α-Syn aggregation-induced toxicity, including proteinase activity inhibition, mitochondrial dysfunction, and cytotoxicity, particularly in the context of the E46K mutation (Jiang et al., 2010).

Baicalein has shown multiple protective effects against α-Syn aggregation. It upregulates autophagy, reduces α-Syn expression, and prevents the sustained accumulation of α-Syn aggregates. Moreover, baicalin inhibits protofibril formation, degrades pre-formed protofibrils, and promotes the structural transformation of α-Syn into larger, soluble aggregates (Li et al., 2017a; Lu et al., 2011). Investigations into the structural characteristics of baicalein-stabilized oligomers have revealed that these oligomers adopt spherical structures with widths of only 8–22 nm. These spherical oligomers exhibit favorable thermodynamic and structural stability, preventing further conversion into fibrillar structures and mitigating their disruptive effects on LM (Hong J. et al., 2008).

Interestingly, baicalein differentially affects the structural integrity and stability of various α-Syn fibrils types. In E46K and H500 mutant fibrils, baicalein binds to both the C-terminal and N-terminal regions. In wild-type fibrils, it exhibits enhanced binding to the NAC region and disrupts the E46-K80 salt bridge. Furthermore, in E46K protofibrils, baicalein disrupts the E61-K80 salt bridge (Yao et al., 2022).

*In vivo* studies have provided further support for the neuroprotective effects of baicalein. In a rotenone-induced mouse model of PD, intraperitoneal (i.p.) administration of baicalein for 7–12 weeks significantly reduced α-Syn aggregation in multiple brain regions. This reduction in α-Syn aggregation was accompanied by the protection of dopaminergic neurons and improvement in behavioral deficits. However, baicalein did not appear to affect α-Syn secretion, as evidenced by the lack of change in α-Syn mRNA expression (Hu et al., 2016). Furthermore, in an MPP^+^-induced PD mouse model, baicalein exerted anti-inflammatory effects, likely mediated through the inhibition of α-Syn aggregation (Hung et al., 2016) (Table 2).

Despite its promising preclinical profile, baicalein suffers from poor water solubility and rapid metabolism, resulting in low bioavailability (13.1%–23.0%). Encouragingly, multiple human trials have demonstrated the safety and tolerability of baicalein within a dosage range of 100–2,800 mg (Pang et al., 2016; Li M. et al., 2014; Li H. et al., 2021). Nanoparticle-based delivery systems have shown promise in overcoming baicalein’s bioavailability limitations. These nanoformulations enhance baicalein’s solubility and stability, leading to improved BBB permeability and enhanced neuroprotection (Aliakbari et al., 2021). Taken together, *in vivo* and *in vitro* studies suggest that baicalein can inhibit various stages of neurotoxic α-Syn production, highlighting its potential as a neuroprotective agent for PD treatment.

### 4.2 Naphthoquinones

Naphthoquinone, a ubiquitous quinonoid organic compound, is characterized by an unsaturated six-carbon ring structure containing two carbonyl groups. It is primarily found in the natural metabolites of plants, animals, fungi, and bacteria. Naphthoquinone exhibits a wide range of biological activities, including antioxidant, anti-inflammatory, antimalarial, antitumor, antibacterial, and neuroprotective effects. Of particular interest, 1,4-naphthoquinone (1,4-NQ) (Figure 6.6) plays a crucial role in maintaining neuronal cell viability, effectively protecting cells against oxidative stress induced by neurotoxins (Santos et al., 2023; Aminin and Polonik, 2020).

**FIGURE 6:**
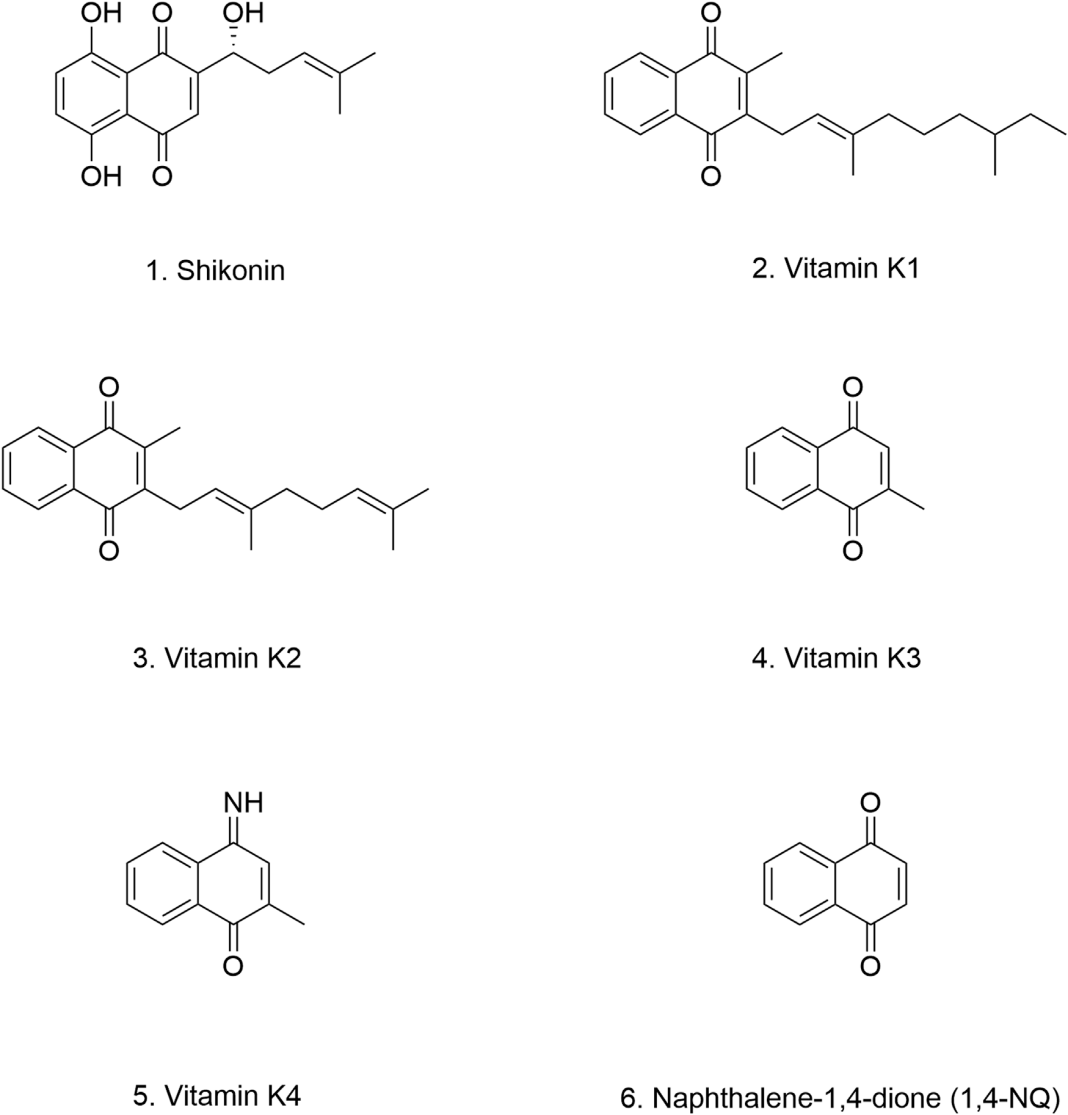
Chemical structure of Naphthoquinones.

#### 4.2.1 Shikonin

Shikonin (SHK) (Figure 6.1) is a bioactive naphthoquinone compound extracted from the roots of *Lithospermum erythrorhizon* (Andújar et al., 2013). It is well-known for its free radical scavenging, antioxidant, and anti-inflammatory properties (Chen X. et al., 2001). Srivastava et al. demonstrated that SHK dose-dependently inhibits α-Syn aggregation both *in vivo* and *in vitro*. Increasing concentrations of SHK prolonged the lag phase of α-Syn aggregation, the lag phase of α-Syn aggregation can be extended to 26.14 h in the presence of equimolar α-Syn and SHK. Mechanistically, SHK binds to the C-terminus of α-Syn, preserving its helical and disordered secondary structures while reducing β-sheet content. This interaction stabilizes α-Syn monomers, reduces the complexity of aggregated structures, and delays fibril elongation. In *C. elegans* model, SHK significantly reduced α-Syn aggregation, improved motor deficits, and attenuated dopaminergic neurons degeneration (Srivastava et al., 2023) (Table 3).

**TABLE 3:** Positive effects of naphthoquinones targeting α-Syn *in vivo* and *in vitro* models of PD.

Natural products	*In vitro* and *in vivo* model	Effects and mechanisms observed	Reference
Shikonin (SHK)	*C. elegans* NL5901 PD model	1.Prevents α-Syn aggregation and accelerates the disaggregation of preformed fibrils, delays elongation of seeded α-Syn aggregation 2.Binds to the C-terminus of α-Syn, maintains α-helical and disorder secondary structures, reduces the β-sheets content and complexity of aggregates 3.Improves movement and rescues dopaminergic neurodegeneration in NL5901	Srivastava et al. (2023)
Vitamins K and 1,4-naphthoquinones (1,4-NQ)	α-Syn aggregation assay	1.Forms smaller, sheared fibrils and amorphous aggregates, which were less capable of inducing vesicle leakage 2.Inhibits α-Syn fibrillization, damages fibrils stability, inhibits fibrils elongation	da Silva et al. (2013)

These neuroprotective effects highlight SHK’s potential as a therapeutic agent for synucleinopathies, including PD. However, SHK’s limited solubility and chemical stability hinder its biological activity. Pharmacokinetic studies have revealed a half-life of 630.7 ± 124.9 min, a maximum concentration of 83.6 ± 8.8 ng/mL, and a time to maximum concentration of 1.0 ± 0.0 min. Importantly, prolonged or rapid administration, particularly at high doses, can lead to hepatotoxicity and nephrotoxicity (Sun et al., 2022).

#### 4.2.2 Vitamin K

Vitamin K, a fat-soluble vitamin found abundantly in green leafy vegetables, encompasses various forms, including Vitamin K_1_ (Figure 6.2), Vitamin K_2_ (Figure 6.3), Vitamin K_3_ (Figure 6.4), Vitamin K_4_ (Figure 6.5), and other forms (Mladěnka et al., 2021). While initially recognized for its essential role in blood clotting (Mishima et al., 2023), recent studies have highlighted the potential of vitamin K, particularly 1,4-NQ (Figure 6.6), in treating neurodegenerative diseases. These therapeutic benefits likely stem from vitamin K’s antioxidant, anti-inflammatory, and anti-demyelination properties (Santos et al., 2023; Emekli-Alturfan and Alturfan, 2023; Diachenko et al., 2024; Sandeep et al., 2023). Fernanda et al. demonstrated the inhibitory effects of vitamin K and 1,4-NQ on α-Syn aggregation. Both compounds effectively decelerated α-Syn fibrillation by interacting with the N-terminal repeat domain of α-Syn monomers. Treatment with vitamin K or 1,4-NQ resulted in the formation of smaller, fragmented fibrils and amorphous aggregates, which exhibited a reduced capacity to induce vesicle leakage (Table 3).

Interestingly, a case-control study found significantly lower serum vitamin K levels in PD patients compared to healthy controls. Moreover, serum vitamin K levels were higher in early-stage PD patients than in those with later-stage disease, suggesting that vitamin K deficiency may contribute to PD pathogenesis and that vitamin K supplementation may hold therapeutic and preventive potential (Liu L. et al., 2023; Yu et al., 2020). Given that inhibiting MAO activity is a key pharmacological target in PD treatment, and based on the structural features of 1,4-NQ, vitamin K, shows promise for developing MAO inhibitors and represents a promising avenue for developing inhibitors of α-Syn fibrillation and aggregation (da Silva et al., 2013).

### 4.3 Tanshinones

*Danshen*, a medicinal plant with a rich history of use in China, is renowned for its protective effects on cardiovascular and cerebrovascular function. Its safety has been well established through centuries of traditional use. Tanshinones, the primary bioactive constituents extracted from the dried roots of *Danshen*, include tanshinone I and tanshinone IIA (TAN I and IIA) (Figures 7.1, 7.2) (Li L. et al., 2021). Despite their therapeutic potential, the bioavailability of tanshinones is limited by their poor water solubility and dissolution rate. However, their lipophilic nature presents opportunities for enhancing their bioavailability through various formulation strategies. For example, compared to other extracts, tanshinone formulations have demonstrated improved maximum concentration and half-life values (Wang D. et al., 2020; Wang X. et al., 2020).

**FIGURE 7:**
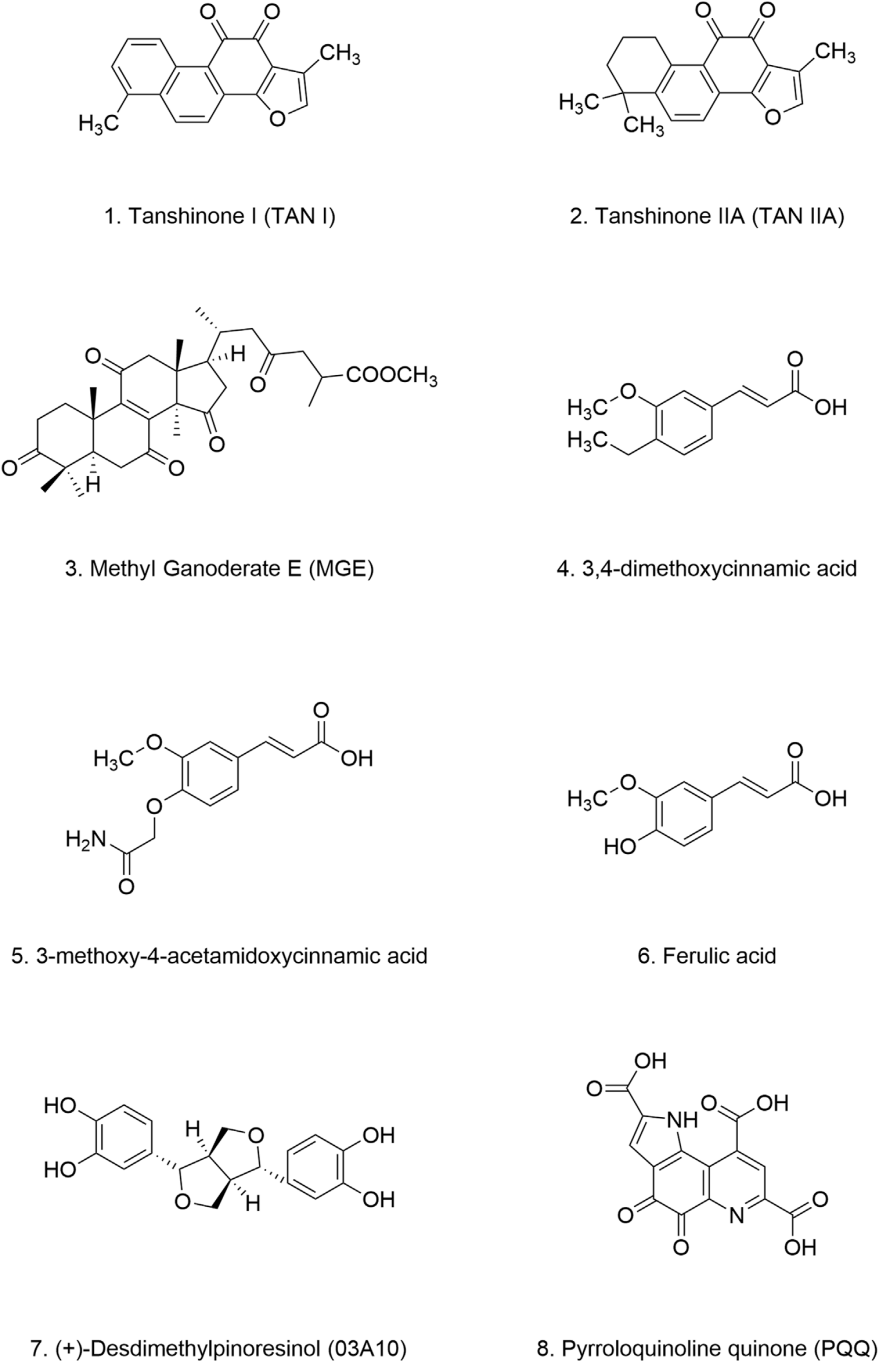
Chemical structure of other natural products.

Both TAN I and IIA have demonstrated remarkable efficacy in inhibiting amyloid-β peptide aggregation and promoting the breakdown of existing amyloid fibrils (Ren et al., 2017; Ren et al., 2015; Wang et al., 2013). *In vitro* experiments have revealed that both compounds can prolong the lag phase of α-Syn aggregation and promote the degradation of preformed, mature α-Syn fibrils, likely by influencing β-sheet formation. Furthermore, treatment with TAN I and TAN IIA significantly extended the lifespan of NL5901, likely due to a reduction in α-Syn aggregation and fibril formation (Ji et al., 2016). (Table 4) These findings underscore the potential of tanshinones as therapeutic agents for neurodegenerative diseases, including PD.

**TABLE 4:** Positive effects of other NPs targeting α-Syn *in vivo* and *in vitro* models of PD.

Natural products	*In vitro* and *in vivo* model	Effects and mechanisms observed	Reference
Tanshinone I and Tanshinone IIA (TAN I and II A)	*In vitro* and transgenic *C. elegans* PD model	1.Affects α-Syn structure transformation, reduces the destruction of LM caused by α-Syn 2.Reduces α-Syn oligomerization and fibrillation, decomposes the pre-formed α-Syn aggregates 3.Prolongs the life span of NL5901	Ji et al. (2016)
Methyl Ganoderate E (MGE)	*C. elegans* PD model	Reduces the accumulation of α-Syn, prolongs the lifespan of NL5901 strains and improves the fertility and locomotion ability	Okoro et al. (2024)
*Centella asiatica* (CA) extract	α-Syn aggregation assay	1.Stabilizes α-Syn monomers, maintains their disordered structure 2.Inhibits the formation of α-Syn aggregates, breaks the preformed α-Syn fibrils	Berrocal et al. (2014)
Cinnamic acid derivatives (CADs)	α-Syn aggregation assay and structure analysis	Binds to α-Syn prefibrillar oligomers or short fibrils, affects the regular structure and inhibits fibrils growth	Medvedeva et al. (2020)
Geum urbanum extract	α-Syn aggregation assay	1.Inhibits α-Syn aggregation 2.Stabilizes α-Syn fibrillation 3.Decomposes preformed α-Syn fibers	Lobbens et al. (2016)
(+)-Desdimethylpinoresinol (03A10)	α-Syn-overexpressing cell lines MPTP-induced mouse model	1.Inhibits the aggregation of α-Syn, reduces the seed toxicity of α-Syn fibrils and prevents the growth of α-Syn fibrils 2.Improves neuronal degeneration, behavioral defects, olfactory dysfunction and intestinal inflammation in mice	Wang Q. et al. (2023)
Pyrroloquinoline quinone (PQQ)	α-Syn aggregation assay U2-OS cells	PQQ, PQQ-α-Syn complex and PQQ-modified α-Syn_36–46_ peptide prevents the formation of α-Syn	Yoshida et al. (2013), Kobayashi et al. (2006)

### 4.4 *Lingzhi* extracts

*Ganoderma lingzhi,* commonly known as *Lingzhi* is a highly valued medicinal mushroom. Its triterpenoids are considered to be key active components contributing to its therapeutic effects (Boh et al., 2007). Numerous studies have demonstrated the efficacy of *Ganoderma* in preclinical models of PD, attributing its beneficial effects to several mechanisms, including selective protection of dopaminergic neurons in the SN, reduction of oxidative stress, preservation of mitochondrial function, mitigation of neuroinflammation, and modulation of neural immunity (Li et al., 2017b; Guo Y. J. et al., 2016; Ren et al., 2019; Zhang et al., 2011).

Methyl Ganoderate E (MGE) (Figure 7.3), a triterpenoid compound isolated from *Lingzhi*, has shown inhibitory effects on the aggregation of both α-Syn and amyloid-β proteins. In NL5901, treatment with MGE at a concentration of 10 μg/mL resulted in a 15% reduction in α-Syn aggregation (Okoro et al., 2024) (Table 4). These findings suggest that *Lingzhi* extracts, particularly its bioactive component MGE, holds promise as a potential therapeutic agent for PD. However, it is important to note that systematic studies on key pharmacological parameters of MGE, such as pharmacokinetics, bioavailability, safety, and efficacy are currently lacking, Further research is warranted to evaluate the clinical potential of MGE for treating PD.

### 4.5 *Centella asiatica* extracts

*Centella asiatica* (CA), commonly known as Gotu Kola, is a traditional herb with a long history of use in Chinese and Ayurvedic medicine (Singh P. K. et al., 2012). *In vitro* studies have confirmed the safety of CA, and it exhibits good BBB permeability without apparent toxic effects. However, the absolute bioavailability of CA is extremely low, only 1.86%, likely due to incomplete absorption and a rapid excretion rate (He et al., 2023). While traditionally used for treating skin conditions, emerging research has revealed the neuroprotective effects of CA. These neuroprotective benefits include enhancing memory, improving cognitive function, and stimulating neuronal growth (Gadahad et al., 2008; Veerendra Kumar and Gupta, 2002).

Extracts from CA have demonstrated promising effects against α-Syn aggregation. Studies have shown that CA extracts can stabilize α-Syn monomers, maintain their disordered structure, inhibit the formation of α-Syn aggregates and even promote the breakdown of preformed α-Syn fibrils, achieving a degradation rate of up to 70% (Berrocal et al., 2014). These protective effects are attributed to the synergistic actions of multiple compounds present in CA extracts, including caffeic acid, chlorogenic acid, gallic acid, and selenium. Furthermore, in a *Drosophila* model of PD, CA extracts significantly delayed motor dysfunction (Siddique et al., 2014). However, a recent study investigating the effects of Ayurvedic nootropics on α-Syn aggregation found that CA extracts, surprisingly, did not exhibit significant inhibitory activity (Anjaneyulu et al., 2020) (Table 4). This discrepancy might be attributed to variations in the composition of CA extracts obtained through different extraction methods. Further research is crucial to identify the specific CA extracts that confer the most potent therapeutic benefits in the context of PD.

### 4.6 Cinnamic acid derivatives (CADs)

CADs have garnered increasing attention due to their structural resemblance to curcumin and their reported antimicrobial, antifungal, anti-inflammatory, anticancer, and neuroprotective properties (Ruwizhi and Aderibigbe, 2020). Found abundantly in plants like *Cinnamomum cassia* and *Panax ginseng*, as well as in fruits, whole grains, and green coffee beans, CADs represent a promising class of bioactive compounds. Mara et al. demonstrated that natural CADs, 3,4-dimethoxycinnamic acid (Figure 7.4), 3-methoxy-4-acetamidoxycinnamic acid (Figure 7.5), and ferulic acid (Figure 7.6), can bind to both α-Syn oligomers and fibrils, effectively reducing β-sheet content within α-Syn aggregates. This interaction prevents the amyloidogenic transformation of α-Syn and ultimately inhibits its aggregation (Medvedeva et al., 2020). Importantly, both naturally extracted and chemically synthesized CADs exhibit these beneficial effects (Table 4). Notably, plasma levels of CADs increase significantly following the consumption of coffee containing CADs (Farrell et al., 2012; Nagy et al., 2011; Medvedeva et al., 2022). Their natural occurrence and presence in human blood, coupled with their potent anti-aggregation properties, highlight the potential of CADs as therapeutic candidates for PD.

### 4.7 *Geum urbanum* extracts

*Geum urbanum* (GU), a member of the *Rosaceae* family, is a medicinal herb with a long history of use in treating ailments such as gastric mucosal and oral inflammation, and it also exhibits cardioprotective properties. Ellagitannins, procyanidins, gallic acid, and vanillic acid constitute the primary components of GU extracts (Neshati et al., 2018; That et al., 2018). Recent studies have revealed that GU extracts not only stabilizes α-Syn fibrils but also promotes their disaggregation in a concentration-dependent manner (Lobbens et al., 2016). At a high concentration (0.25 mg/mL), the extracts inhibited fibril formation for up to 40 h, with the lag phase of fibrillation increasing proportionally with extracts concentration. Remarkably, the ability of α-Syn to form fibrils was attenuated to varying degrees under all tested conditions, with earlier addition of the extracts yielding more pronounced inhibitory effects. This observation suggests that GU extracts may reduce the fibrillation and aggregation propensity of one or more intermediate oligomeric species, thereby delaying α-Syn aggregation (Lobbens et al., 2016) (Table 4). However, due to the complex composition of the extracts, the precise structural characteristics and structure-function relationships of the active components remain to be elucidated. Moreover, the safety profiles of the derived oligomers warrant further investigation. Nevertheless, the strategy of targeting multiple intermediate species to mitigate their fibrillation potential represents a novel approach for developing disease-modifying therapies for PD.

### 4.8 (+)-Desdimethylpinoresinol

(+)-Desdimethylpinoresinol (03A10) (Figure 7.7), originally identified in the fruit of *Vernicia fordii*, has emerged as a potent inhibitor of α-Syn aggregation. *In vitro* studies have demonstrated that 03A10 does not significantly affect cell viability, supporting its safety profile as a potential therapeutic agent. Treatment with 03A10 resulted in a tenfold reduction in the size of α-Syn fibrils, accompanied by a decreased rate of aggregate formation and inhibition of β-sheet formation. Interestingly, 03A10 exhibited no appreciable affinity for α-Syn monomers, suggesting that its mechanism of action does not involve direct interaction with the monomeric form. Instead, 03A10 preferentially bound to α-Syn fibrils and displayed a high affinity for α-Syn aggregates. In a mouse model of PD, oral administration of 03A10 led to varying degrees of improvement in α-Syn aggregation and propagation, neuronal degeneration, behavioral deficits, olfactory dysfunction, and intestinal inflammation (Wang Q. et al., 2023) (Table 4).

### 4.9 Natural alkaloid compounds

Natural alkaloids, a class of nitrogen-containing organic compounds ubiquitous in plants, derive their name from their characteristically alkaline nature. These compounds exert a wide range of pharmacological effects, primarily by binding to specific receptors within biological systems. The remarkable diversity of their biological activities and pharmacological properties has garnered significant attention from researchers across various disciplines.

#### 4.9.1 Quinoline and indole alkaloids

Natural alkaloids represent a promising source of bioactive compounds for modulating α-Syn aggregation. In a study investigating the relationship between alkaloids and α-Syn aggregation, six out of nine alkaloids tested (Figures 8.1–8.6), all derived from medicinal herbs, exhibited inhibitory effects on α-Syn seed fibril formation and seed-induced toxicity (Ghanem et al., 2021). Similarly, previous studies have shown that 2-(quinoline-8-carboxamido)benzoic acid (2-QBA) (Figure 8.8), a natural quinoline alkaloid isolated from *Aspergillus* sp. SCSIO06786, enhances proteasome activity, reduces the toxicity associated with α-Syn aggregates, and ameliorates motor dysfunction in a nematode model of PD (Lee et al., 2023). Moreover, Harmine (Figure 8.4), a β-carboline alkaloid found in *Peganum harmala*, and Lycorine (Figure 8.7), an isoquinoline alkaloid extracted from *Amaryllidaceae* plants, acts as natural enhancers of the UPS, can significantly enhance the phosphorylation of PKA, thereby activating the UPS to promote the degradation of pathogenic α-Syn in both *in vitro* and *in vivo* models of PD (Zhu et al., 2021; Cai et al., 2019). Furthermore, tetracyclic oxindole alkaloids, such as isorhynchophylline (Figure 8.9), corynoxine B, and corynoxine (an enantiomer of corynoxine B) (Figure 8.10), isolated from the Chinese herbal medicine *Uncaria rhynchophylla*, function as natural autophagy inducers. These alkaloids have been shown to mitigate α-Syn aggregation by inducing autophagy (Chen et al., 2014; Lu et al., 2012). Corynoxine B acts through the autophagy-regulating factor Beclin 1, whereas corynoxine exerts its effects through the AKT/mTOR pathway (Table 5). These findings provide compelling evidence for the potential of natural alkaloids as valuable leads for developing novel autophagy enhancers for the treatment of PD.

**FIGURE 8:**
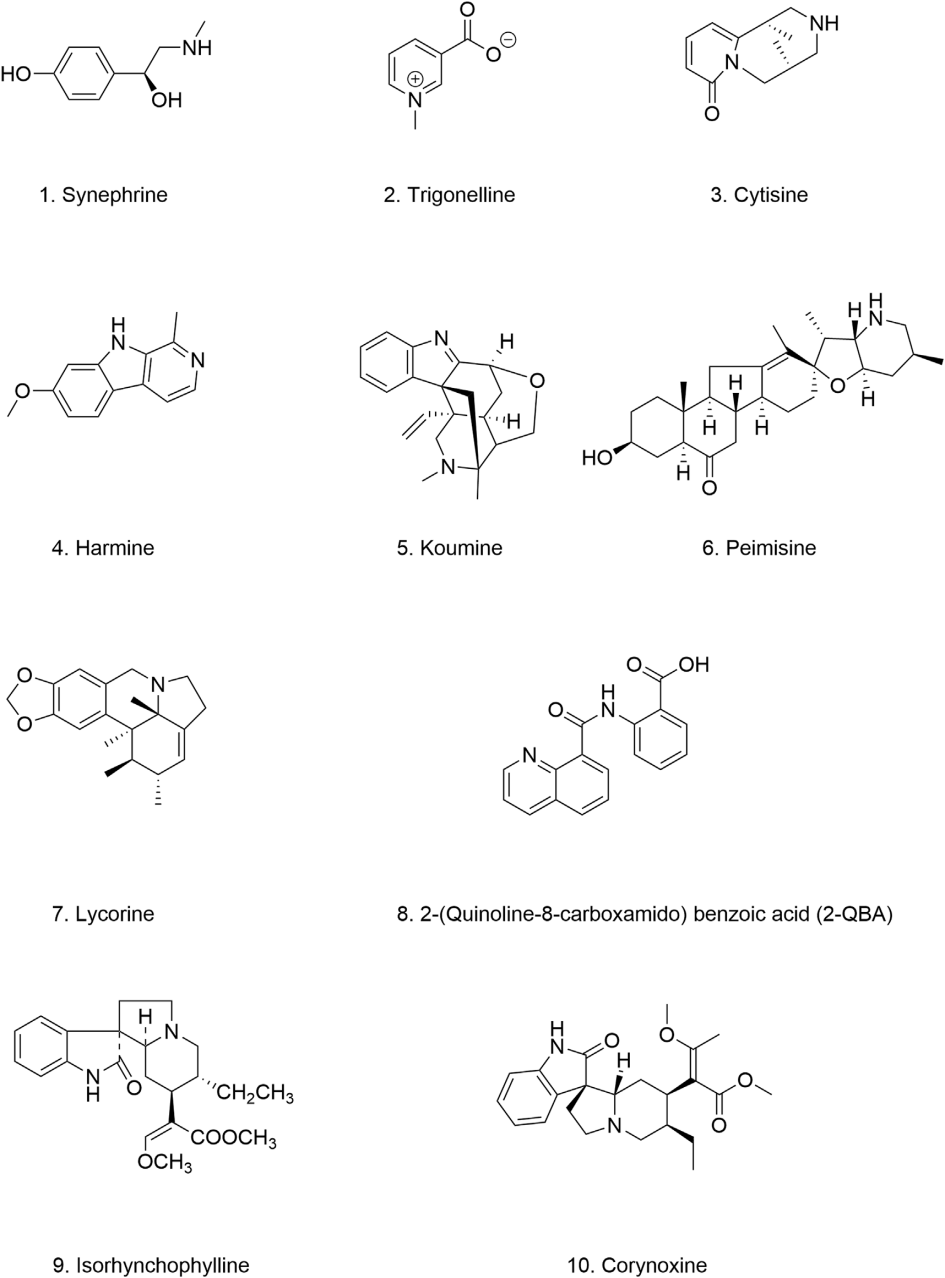
Chemical structure of quinoline and indole alkaloids.

**TABLE 5:** Positive effects of natural alkaloid compounds targeting α-Syn *in vivo* and *in vitro* models of PD.

Natural products	*In vitro* and *in vivo* model	Effects and mechanisms observed	Reference
Synephrine, Trigonelline, Cytisine, Harmine, Koumine, Peimisine	α-Syn aggregation assay	Inhibits α-Syn-seeded fibrils formation and reduces α-Syn-seeding-dependent toxicity	Ghanem et al. (2021)
Harmine, Lycorine	α-Syn overexpression cell and mice model	1. Enhances PKA phosphorylation to enhance UPS function, promoting α-Syn degradation and clearance 2. Rescues cell death induced by α-Syn overexpression	Zhu et al. (2021), Cai et al. (2019)
2-(Quinoline-8-carboxamido) benzoic acid (2-QBA)	*C. elegans* PD model	1. Inhibits the expression of α-Syn, enhances the activity of the proteasome, and regulates the formation of α-Syn oligomers 2. Improves MPP^+^-induced neurodegeneration and improves NL5901 behavior disorders	Lee et al. (2023)
Isorhynchophylline, Corynoxine B and Corynoxine	N2a and SH-SY5Y cells, PC12 cells and Primary neuron *Drosophila*	As a natural inducer of autophagy, induces autophagy in different neuronal lines, reduces the erroneous aggregation of α-Syn	Chen et al. (2014), Lu et al. (2012)
Nicotine	α-Syn aggregation assay	1. Inhibits the α-Syn formation 2. Inhibits α-Syn fibrillation and stabilizes soluble oligomeric forms 3. Destabilizes preformed α-Syn fibrillation	Ono et al. (2007), Hong et al. (2009)
	α-Syn aggregation assay and structural analysis	Interacts with all α-Syn domains with van der Waals Force, slows down the conformational transition of α-Syn	Alıcı (2021)
	α-Syn aggregation assay	1. Increases the lag phase of the nucleation and reduces the build-up of the oligomers 2. Binds to and induces conformational change in monomeric α-Syn, reduces the accumulation of aggregates 3. Reduces the oxidative stress in the cell, cytotoxicity and increases cell survival	Kardani et al. (2017)
	*Drosophila*	The effects such as improvement of dyskinesia, neuroprotection, stabilization of α-Syn, inhibition of the aggregation of α-Syn aggregation, are associated with synaptic vesicle glycoprotein	Olsen et al. (2023)
Caffeine	α-Syn aggregation assay Yeast cells expressing WT α-Syn	1. Changes α-Syn properties, accelerates α-Syn nucleation and fibrillation rate, reduces oligomers and aggregates toxicity 2. Maintains the retain the lipid-binding properties of aggregates, reduces cellular oxidative stress and cell damage	Kardani and Roy (2015)
	mouse striatum with A53T α-Syn fibers	1. Chronic caffeine treatment reduces a series of pathological changes such as pSer129α-Syn-rich aggregates, apoptotic neuronal cell death, microglia, and astroglia reactivation 2. Selectively reverses α-Syn-induced defects in macro-autophagy and CMA induced by A53T α-Syn fibrils	Luan et al. (2018)
Squalamine	α-syn aggregation assay SH-SY5Y cells Overexpression of α-syn in the muscle cells of the nematode worms	1. Displaces α-Syn from LM, inhibits abnormal α-Syn aggregation 2. Competitive binds membrane surface sites, decreases α-helix content of α-syn 3. Markedly decreases the mitochondrial damage, the increase of intracellular ROS level and toxicity caused by α-Syn 4. Improves motility of the PD worms	Perni et al. (2017)
Trodusquemine	α-syn aggregation assay PD worms expressing α-syn	1. Displaces α-Syn from LM, inhibits abnormal α-Syn aggregation and secondary nucleation 2. Binds to α-Syn fibrils, preventing fibrils proliferation and aggregates formation 3. Suppresses the toxicity of α-Syn 4. Increases both fitness and longevity of the PD Worms	Perni et al. (2018)

#### 4.9.2 Nicotine

Epidemiological studies have consistently revealed a negative correlation between smoking and the incidence of PD (Quik, 2004). Nicotine (Figure 9.1), the primary alkaloid found in *Solanaceae* plants, has been shown to exert neuroprotective effects through the activation of nicotinic receptors located at dopaminergic terminals, which modulates DA release (Ma et al., 2017; Quik et al., 2012).

**FIGURE 9:**
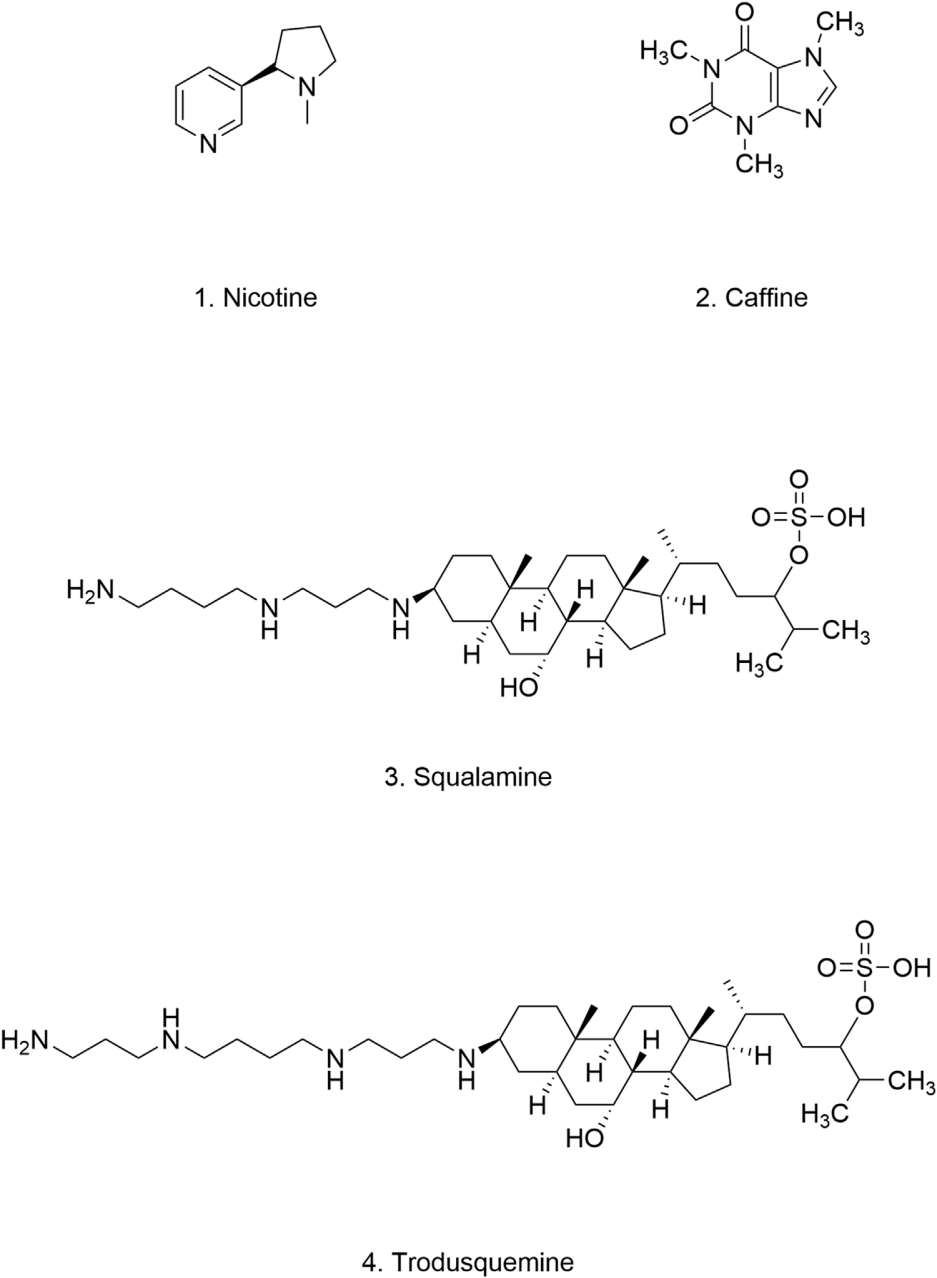
Chemical structure of nicotine, caffeine, squalamine and trodusquemine.

Studies suggest that nicotine can influence α-Syn dynamics and potentially modulate PD pathogenesis. Nicotine has been shown to stabilize soluble α-Syn oligomers, thereby inhibiting the formation of α-Syn fibrils and reducing the instability of preformed fibrils (Ono et al., 2007; Hong et al., 2009). It interacts extensively with α-Syn, engaging nearly all of its structural domains through van der Waals forces. This interaction helps maintain the helical structure of α-Syn and prevents detrimental conformational changes (Tavassoly et al., 2014; Alıcı, 2021). Moreover, nicotine can prolong the rate of aggregate nucleation, effectively delaying the conversion of α-Syn monomers into oligomers (Kardani et al., 2017). Interestingly, studies using synaptic vesicle glycoprotein 2 knockdown in *Drosophila* models suggest that nicotine may influence PD pathology by modulating vesicle release (Olsen et al., 2023) (Table 5). Collectively, these findings highlight the therapeutic potential of nicotine, and other agents targeting nicotinic acetylcholine receptors, as potential disease-modifying agents for PD.

Smoking represents the most rapid and efficient route for delivering nicotine to the bloodstream and brain, achieving stable concentrations within 30 min of intake. Due to the first-pass metabolism of the liver, the bioavailability of oral nicotine is about 20%–45%, while the bioavailability through inhalation is substantially higher (Hukkanen et al., 2005). While nicotine exhibits promising neuroprotective properties, its delivery via smoking is inextricably linked to the detrimental effects of tobacco smoke. The addictive and stimulant properties of nicotine, primarily attributed to its action on nicotine acetylcholine receptors, necessitate a cautious approach to its therapeutic application. This is particularly relevant for individuals with PD, especially elderly patients who may be more susceptible to nicotine addiction. Therefore, determining the effective dosage, optimal route of administration, and long-term safety of nicotine for PD treatment remains a significant challenge that warrants further investigation.

#### 4.9.3 Caffeine

Caffeine (Figure 9.2), a xanthine alkaloid primarily derived from coffee, tea, and cocoa, has been linked to a reduced incidence of PD (Qi and Li, 2014). As a non-selective adenosine A_2A_ receptor antagonist, caffeine may exert its neuroprotective effects on dopaminergic neurons by inhibiting A_2A_ receptor signaling pathways (Ren and Chen, 2020; Qi and Li, 2014; Chen J. F. et al., 2001). Several studies have explored the intricate relationship between caffeine and α-Syn. Caffeine has been shown to induce conformational changes in α-Syn by binding to its N-terminal and C-terminal regions, thereby hindering its aggregation and preserving its lipid-binding properties (Tavassoly et al., 2014). Intriguingly, caffeine appears to modulate the aggregation kinetics of α-Syn in a complex manner. It has been reported to increase the formation of α-Syn oligomers and accelerate the conversion of oligomers into fibrils, ultimately leading to the formation of new, stable fibrils with reduced oligomer presence. This observation is significant because these newly formed fibrils exhibit lower toxicity compared to their oligomeric counterparts (Kardani and Roy, 2015). Furthermore, caffeine has demonstrated autophagy-enhancing properties. Chronic caffeine treatment has been shown to rescue macroautophagy defects induced by the A53T α-Syn mutation, accelerate the clearance and degradation of α-Syn, and alleviate α-Syn fibril-induced apoptosis in mice (Luan et al., 2018) (Table 5).

Moreover, caffeine’s dual solubility in water and lipids, along with its high BBB permeability, makes it a promising therapeutic agent for brain disorders. With an absorption rate of up to 99% in the gastrointestinal tract, a half-life of 2.5–4.5 h, and good oral bioavailability and tolerance, caffeine demonstrates favorable pharmacological properties (Fredholm et al., 1999). These characteristics, coupled with its ability to enhance autophagy and modulate α-Syn aggregation, suggest potential for targeted therapy in PD. However, despite its generally safe profile, excessive caffeine intake (exceeding grams) can lead to adverse effects, even fatal consequences, especially in individuals with certain metabolic disorders, liver diseases, or cardiovascular conditions (Musgrave et al., 2016). Therefore, careful evaluation of caffeine’s optimal dosage, adverse effects, and long-term toxicity remains crucial.

#### 4.9.4 Squalamine

Squalamine (Figure 9.3), a water-soluble aminosterol isolated from the dogfish shark (*Squalus acanthias*), is well-known for its potent antibacterial properties (Moore et al., 1993). As a cationic amphipathic steroid, squalamine exhibits a high affinity for phospholipid membranes containing negatively charged headgroups (Selinsky et al., 1998; Brunel et al., 2005). Michele et al. demonstrated *in vitro* that squalamine’s positive charge mediates strong binding to anionic groups on LM, thereby inducing the dissociation of α-Syn from the LM. This competitive interaction effectively blocks α-Syn binding sites on the LM, inhibiting the formation of α-Syn aggregates. Furthermore, in a *C. elegans* model of α-Syn, squalamine treatment significantly reduced the cytotoxicity associated with α-Syn aggregates and ameliorated the behavioral and motor deficits (Perni et al., 2017). These findings suggest that squalamine inhibits α-Syn aggregation by targeting the kinetics of the aggregation process, a mechanism distinct from the previously reported inhibitory effects of NPs.

Squalamine has emerged as a promising therapeutic candidate for PD. Notably, ENT-01 (patent: CN106535902), the first squalamine-based drug, entered human clinical trials for PD in 2018 (NCT03781791). Phase II results indicated that ENT-01 exhibits a favorable safety profile, and 80% of patients experienced significant relief from constipation, a common NMS of PD, following ENT-01 treatment. Further evaluations of ENT-01’s effects on other neurological symptoms are planned for future studies (Hauser et al., 2019). Previous studies have provided evidence supporting the efficacy of squalamine in PD treatment, with preliminary findings suggesting that an oral dose of 500 mg/m^2^/day is relatively safe. However, further studies are needed to assess the potential for adverse effects associated with long-term or frequent squalamine administration (Hao et al., 2003).

#### 4.9.5 Trodusquemine

Like squalamine, trodusquemine (Figure 9.4) is an aminosterol isolated from the liver of *Squalus acanthias*. Structurally similar to squalamine (Rao et al., 2000), trodusquemine also exerts inhibitory effects on α-Syn aggregation by displacing it from LM. However, in addition to inhibiting the initial nucleation event, trodusquemine can directly bind to preformed α-Syn fibrils, thereby preventing amplification of aggregation through secondary nucleation. This dual inhibition of nucleation processes may confer superior cytoprotective properties to trodusquemine compared to squalamine (Perni et al., 2018). Although experimental data regarding its pharmacokinetics, bioavailability, safety, and toxicological profile are currently limited, trodusquemine’s reported ability to penetrate the BBB and promote tissue regeneration makes it an attractive candidate for treating synucleinopathies, including PD.

### 4.10 Pyrroloquinoline quinone

Pyrroloquinoline quinone (PQQ) (Figure 7.8) is a potent antioxidant nutrient mainly synthesized by Gram-negative bacteria. It is naturally present in various foods, including natto, celery, tofu, kiwi, and oolong tea. Both animal and human studies have demonstrated the safety and tolerability of PQQ as an antioxidant and nutritional supplement. PQQ exhibits high oral absorption (62%) and does not accumulate significantly in the body. Moreover, no notable toxic effects have been reported (Akagawa et al., 2016).

PQQ has demonstrated inhibitory effects on amyloid formation, including that of α-Syn, β-amyloid protein, and mouse prion protein (Kim et al., 2010). Kobayashi et al. revealed through *in vitro* experiments that PQQ can inhibit α-Syn aggregation in a dose-dependent manner by forming a PQQ-α-Syn complex via a Schiff base structure (Kobayashi et al., 2006). Subsequently, the same group identified the α-Syn_36-46_ peptide as the key sequence responsible for the inhibitory effect of the PQQ–α-Syn complex. Interestingly, modifications of the α-Syn_36-46_ peptide with other compounds possessing quinone structures, such as curcumin and EGCG, also prevented α-Syn aggregation and fibril formation (Yoshida et al., 2013) (Table 4). These findings suggest that targeting the α-Syn_36–46_ peptide with specifically structured compounds, particularly those containing quinone moieties, represents a promising strategy for developing α-Syn aggregation inhibitors. While PQQ itself exhibits suboptimal BBB permeability, esterification of PQQ can effectively enhance its brain penetration. For example, PQQ-trimethylester, an esterified derivative of PQQ, has been shown to possess twice the BBB permeability of PQQ and exhibits a more potent inhibitory effect on α-Syn aggregation (Tsukakoshi et al., 2018).

## 5 Combined clinical trials and preclinical studies

Currently, clinical data on the use of NPs for the treatment or adjunctive treatment of PD remains limited. Clinical trials investigating the efficacy and safety of several NPs in PD, including curcumin (IRCT20191231045968N1, IRCT20101209005352N2, IRCT20171123037600N1), *Ganoderma lucidum* extracts (ChiCTR2100050538), nicotine (IRCT20210618051612N1, NCT01216904, NCT00873392, NCT01560754, NCT00957918, NCT02452125), and caffeine (NCT01190735, NCT00459420, NCT01738178), have been registered on clinical trials platforms (e.g., trialsearch. who.int, chictr. org.cn, clinicaltrials.gov). However, it is important to note that not all registered trials have been completed.

Clinical trials evaluating curcumin as a potential therapeutic agent for PD have yielded mixed results. Donadio et al. reported that curcumin supplementation (in combination with levodopa or DA agonists) in 21 PD patients led to significant improvements in dyskinesia and NMS, as well as a reduction in α-Syn deposition in the skin (Donadio et al., 2022). In contrast, a separate study involving 30 PD patients found that administration of curcumin nanomicelles for 9 months did not result in significant improvements in dyskinesia or NMS or quality of life (IRCT20171123037600N1) (Ghodsi et al., 2022).

Clinical trials exploring the therapeutic potential of nicotine in PD have produced conflicting results, likely due to variations in dosage and treatment duration. A study employing nicotine patches (maximum dose of 28 mg/24 h) for 52 weeks in individuals with early-stage PD (NCT01560754) did not find support for a beneficial effect of nicotine on disease progression (Oertel et al., 2023). Similarly, another using a comparable nicotine regimen (NCT00873392) reported similar findings (Villafane et al., 2018; Lemay et al., 2004).

In contrast, long-term maintenance therapy with higher doses of nicotine (more than 45–90 mg/day) has shown promise in improving motor function, reducing the need for dopaminergic medication, and potentially slowing the loss of DA transporters (Itti et al., 2009; Villafane et al., 2007). Furthermore, nicotine has consistently demonstrated a positive effect on semantic processing in PD patients, possibly due to its stimulatory effects on DA signaling and enhancement of inhibitory mechanisms (Holmes et al., 2011a; Holmes et al., 2011b). Interestingly, nicotine has also shown potential in managing hypotension, a potentially life-threatening complication of PD. A study investigating the effects of oral nicotine gum (NCT02452125) found that it effectively increased blood pressure in PD patients experiencing acute hypotension (DiFrancisco-Donoghue et al., 2019). These findings suggest that nicotine therapy may represent a novel treatment strategy for specific aspects of PD, including motor symptoms, cognitive impairment, and hypotension. A placebo-controlled, double-blind, randomized trial involving 24 PD patients found that a 100 mg dose of caffeine modestly improved attention (Sharma et al., 2022). Caffeine intake has also been associated with improvements in freezing of gait (Kitagawa et al., 2007). However, caffeine has not consistently demonstrated relief from NMS such as excessive daytime sleepiness (Postuma et al., 2012b). Similarly, two other clinical studies failed to show positive effects of caffeine therapy on motor symptoms or disease progression (Postuma et al., 2017; Simon et al., 2008).

A double-blind, randomized, cross-over trial involving 12 idiopathic PD patients revealed that caffeine accelerates levodopa uptake, significantly shortening the latency period for motor responses (Kyaw et al., 2013). This suggests that caffeine administration prior to levodopa intake may enhance the drug’s efficacy. Supporting this notion, a larger study (n = 222) found that caffeine use was associated with a reduced likelihood of developing motor complications in patients taking pramipexole and levodopa in PD (Wills et al., 2013). In summary, while caffeine exhibits certain positive effects in PD, including modest cognitive enhancement, potential benefits for freezing gait, and modulation of levodopa pharmacokinetics, its overall impact remains complex and likely varies among individuals.

Co-administration of certain natural products with conventional PD medications has shown potential in enhancing therapeutic outcomes. For instance, naringenin has been shown to significantly increase the maximum plasms concentration, brain concentration, and elimination half-life of rasagiline, a commonly prescribed DA agonist, when administered concurrently. Naringenin also reduces the clearance rate of rasagiline, likely due to its inhibitory effects on cytochrome P-450 1A2 (CYP1A2) metabolism (Pingili et al., 2016). Furthermore, naringenin itself exhibits MAO inhibitory activity, suggesting potential neuroprotective properties (Carradori et al., 2016). Similarly, theophylline has been shown to prolong the duration of levodopa’s effects and improve acute “off” periods in individuals with PD (Kulisevsky et al., 2002).

In a gait analysis experiment using a mouse model of PD, co-administration of baicalein with a low-dose of levodopa significantly improved gait disturbances, achieving therapeutic effects comparable to those observed with a high-dose of levodopa alone (Zheng et al., 2019). This suggests that baicalein may enhance the efficacy of levodopa, potentially allowing for lower, better-tolerated doses. Similarly, resveratrol has been shown to alleviate levodopa-induced motor dysfunction in rats without diminishing its anti-PD effects (Zheng et al., 2021).

Several NPs, including baicalein, curcumin, resveratrol, and EGCG, have also exhibited neuroprotective properties against the potential neurotoxicity associated with high-dose levodopa (Kang et al., 2013; Takeshima et al., 2011). These findings suggest that combination therapy with NPs may offer a multifaceted approach to PD treatment by enhancing the efficacy of conventional medications, reducing required doses, and providing neuroprotection. Further research is warranted to optimize these combination strategies and translate them into clinical practice.

## 6 Limitations of preclinical studies and future challenges

Preclinical studies have provided compelling evidence for the neuroprotective properties of natural products against α-Syn aggregation and deposition. However, several factors, particularly those related to pharmacokinetics, limit their clinical translation. Many NPs, including curcumin and resveratrol, suffer from poor bioavailability due to low intestinal absorption rates and extensive first-pass metabolism, which can result in the formation of inactive metabolites. Furthermore, their distribution to the CNS is often restricted by limited BBB permeability. Fortunately, strategies such as structural modification, formulation optimization, and advanced drug delivery systems (e.g., nanotechnology, drug carriers, micelles and cocrystals) have shown promise in overcoming these limitations by significantly improving bioavailability and BBB permeability (Liu M. et al., 2020; Mehmood et al., 2022; Rong et al., 2023; Xing et al., 2017).

Moreover, the inherent complexity and diversity of NPs structures pose challenges for isolating and extracting the specific active constituents responsible for the desired therapeutic effects. The lack of unified and standardized extraction and purification processes can lead to significant batch-to-batch variations in composition and activity, potentially impacting the efficacy and safety of NPs-based therapies. Therefore, establishing standardized protocols for NPs extraction and preparation is crucial for ensuring consistent product quality and facilitating clinical translation. Such standardization would enable more reliable assessment of efficacy and safety in clinical trials and support the development of well-defined, efficacious, and safe NPs-based therapeutics.

While our study highlights the therapeutic potential of NPs in mitigating abnormal α-Syn deposition, a significant knowledge gap exists regarding their precise mechanisms of action. Most studies have primarily focused on demonstrating a reduction in α-Syn aggregation, without delving into the underlying molecular mechanisms. Moreover, α-Syn aggregation is a dynamic, multi-step process, and current detection methods often fall short of capturing the entire aggregation and disaggregation process in real-time. This lack of in-depth mechanistic understanding and limitations in monitoring techniques hinder comprehensive assessment of the specific roles NPs play in inhibiting α-Syn aggregation.

In conclusion, the successful clinical translation of NPs for treating synucleinopathies like PD requires a multifaceted approach. Rigorous preclinical studies are essential for elucidating the precise molecular mechanisms of action, optimizing NPs formulations to enhance bioavailability and BBB permeability, and establishing standardized extraction and preparation processes to ensure product consistency. Furthermore, well-designed clinical trials are crucial for evaluating the therapeutic efficacy and safety of these NPs in humans. These trials should carefully consider appropriate dosage and administration routes, individual patient variability, long-term safety profiles, and potential pharmacological and toxicological interactions with other medications commonly prescribed for PD. Addressing these challenges will pave the way for harnessing the therapeutic potential of NPs and developing effective treatments for synucleinopathies.

Additionally, current research on NPs for PD relies heavily on preclinical models, including toxin-induced models (e.g., MPTP, 6-OHDA, paraquat), transgenic animal and cell models, and simpler organisms like *C. elegans*, *Drosophila*, and zebrafish. These models have proven valuable for studying specific aspects of PD pathogenesis, such as α-Syn misfolding, oxidative stress, mitochondrial dysfunction, and neuroinflammation. Studies utilizing these models have shown that NPs can modulate these pathological processes to varying degrees, leading to improvements in motor dysfunction. However, it is essential to recognize that no single model can fully recapitulate the complex PD phenotype observed in humans. Several factors contribute to the challenge of translating preclinical findings on NPs for PD into clinical applications. Species differences, variations in cellular microenvironments, and discrepancies between *in vitro* and *in vivo* conditions can all limit the translatability of preclinical data to humans. Moreover, inconsistencies in experimental design, such as variations in drug dosages, administration routes, treatment durations, and the lack of standardized outcome measures, can lead to conflicting results across studies, hindering the comparability and reliability of the findings. Research on the therapeutic effects of NPs for PD remains largely confined to preclinical cellular or animal models, with limited clinical data available to support their efficacy in humans. The scarcity of well-designed clinical trials represents a significant obstacle to advancing NPs-based therapies for PD.

## 7 Conclusion

The abnormal aggregation of α-Syn, culminating in the formation of Lewy bodies, represents a hallmark neuropathological feature of PD. The pervasive presence of α-Syn aggregates across diverse brain regions underscores the critical role of α-Syn in the pathogenesis of PD. The efficacy of current PD medications often diminishes over time as treatment duration increases and complications arise. This highlights the urgent need for novel therapeutic strategies that target the underlying mechanisms of disease onset and progression. NPs, derived from various sources in nature, are increasingly recognized as valuable sources of bioactive compounds for drug development and therapeutic applications. Recent research has unveiled a wide array of biological activities associated with NPs, including antioxidant, anti-inflammatory, antitumor, antibacterial, and immunomodulatory effects. Compared to synthetic drugs, NPs often exhibit greater structural complexity and exert their effects through multi-target mechanisms of action. This inherent characteristic of NPs often translates to a more favorable side effect profile, making them particularly attractive for treating complex diseases like PD, which involve intricate and interconnected pathological pathways.

Our study highlights the potential of various NPs in inhibiting the misfolding and abnormal aggregation of α-Syn (Figure 2B). Polyphenols, flavonoids, naphthoquinones, cinnamic acid, CA extracts, and natural alkaloids have emerged as promising candidates for PD therapy. These natural compounds exert anti-aggregation effects through multiple mechanisms, including slowing the nucleation rate of α-Syn, inhibiting oligomer formation, binding to intermediates to delay aggregate formation, destabilizing preformed α-Syn fibrils, and promoting the degradation and clearance of aggregates and fibrils. These multifaceted anti-aggregation properties of NPs offer a promising avenue for the prevention and treatment of PD.

MAO and COMT are important therapeutic targets for PD. Research has confirmed that curcumin, DHM, baicalein and EGCG exhibit potential in inhibiting COMT, while rutin, CAD, tanshinones, SHK, and CA extracts have shown potential in inhibiting MAO (Zheng et al., 2021; Kang et al., 2013; Prajapati et al., 2021; Takao et al., 2017; Zhao et al., 2021; Zhu and Jia, 2014), mean that new CMOT and MAO inhibitors can be developed and utilized on this basis.

We analyzed the existing clinical trials on the use of NPs for treating PD. Some studies have shown positive effects of Curcumin, nicotine, and caffeine, while others are negative. These discrepancies may be due to several factors, including the low absorption and metabolic efficiency, poor BBB permeability of many NPs in the body, or the use of inadequate doses and inappropriate administration methods in clinical trials, all of which can affect the bioactivity of the drugs and result in insufficient concentrations of active compounds to achieve the desired therapeutic effects. Additionally, in clinical settings, individual patient differences, experimental design, insufficient sample sizes, and inconsistent assessment methods can all impact the reliability and validity of results. And short-term observations may fail to capture significant therapeutic effects. Furthermore, multiple studies combining NPs with levodopa have shown that these supplements can effectively optimize levodopa’s efficacy and mitigate its side effects, particularly in cases of fluctuating clinical responses. Supplementation with polyphenolic compounds like naringin and curcumin at the onset of levodopa treatment shows promise as a strategy to potentially mitigate α-Syn aggregation and disease progression. Importantly, common structural motifs, such as quinones (six-membered rings with two double bonds and two ketones) and aromatic rings with hydroxyl groups, have been identified in several effective NPs (Caruana et al., 2011). These structural features appear to be crucial for inhibiting α-Syn aggregation and fibrillation, suggesting that they represent key pharmacophores for the development of novel α-Syn inhibitors. Further exploration of these structural motifs could provide valuable insights and guide drug discovery efforts for PD.

Emerging evidence suggests that therapeutic strategies targeting a single pathway may be insufficient for effectively treating complex diseases like PD. NPs, with their inherent multi-target pharmacological properties and neuroprotective effects, offer a promising alternative by potentially modulating multiple pathological pathways involved in PD pathogenesis. While the natural origin of NPs generally confers a degree of safety, several challenges hinder their clinical translation for treating neurodegenerative diseases like PD. Technical hurdles in extraction, purification, and structural characterization can impact standardization and quality control of NPs-based therapies. Furthermore, research on the pharmacokinetic properties, bioavailability, long-term safety, and potential toxicity of NPs in humans remains limited. Additionally, the BBB poses a significant obstacle to the delivery of many NPs to the brain, limiting their therapeutic efficacy. Developing novel strategies to enhance BBB permeability and facilitate efficient brain delivery of NPs and their bioactive constituents is crucial. Future research should focus on the neurobiological mechanisms by which NPs inhibit α-Syn aggregation and modulate pathways involved in neurodegeneration. Besides, conducting well-designed clinical trials to evaluate the safety, efficacy, optimal dosages, and long-term effects of promising NPs-based therapies in PD patients is needed for unlocking the full therapeutic potential of NPs and developing effective treatments for PD and other neurodegenerative disorders.
